# Thermal reaction norms can surmount evolutionary constraints: comparative evidence across leaf beetle species

**DOI:** 10.1002/ece3.2231

**Published:** 2016-06-12

**Authors:** Dmitry Kutcherov

**Affiliations:** ^1^ Department of Entomology St. Petersburg State University 7‐9 Universitetskaya nab. St. Petersburg 199034 Russia

**Keywords:** Correlation, developmental rate, optimum, phylogenetic comparative methods, temperature threshold, thermal reaction norm

## Abstract

One of the leitmotifs of the ecophysiological research on ectotherms is the variation and evolution of thermal reaction norms for biological rates. This long‐standing issue is crucial both for our understanding of life‐history diversification and for predicting the phenology of economically important species. A number of properties of the organism's thermal phenotype have been identified as potential constraints on the evolution of the rate–temperature relationship. This comparative study addresses several such constraints by testing whether the actual interspecific variation of thermal reaction norms across nearly hundred leaf beetle species agrees with the expected patterns. The results show that developmental rate and its temperature‐dependent parameters are similar in closely related species and that the variation pattern depends on the taxonomic scale, the thermal reaction norms being mostly parallel for the representatives of distant subclades but intersecting more often farther down the phylogenetic tree. The parallel shift disagrees with the putative ubiquity of a positive slope–threshold relationship, whereby thermal reaction norms should normally intersect, and even more contradicts with the common‐intersection hypothesis. The ability to develop in cooler conditions is not traded off at higher temperatures, which is an exception to the “warmer is better” principle. A comparison of high‐ and low‐quality data indicates that some of these discrepancies with earlier findings may stem from a likely presence of noise in previous analyses, which may have affected the variation patterns observed. Overall, the failure to support the universality of the predicted patterns suggests that the evolution of thermal reaction norms in leaf beetles has largely overcome the hypothesized constraints.

## Introduction

The diversity of life is immense in many aspects, and one of these is the astonishing variability of the time spans required by different organisms to develop from a spore or zygote into an adult. Many fruit flies, thrips, and aphids produce multiple annual generations, especially in tropical regions, in impressive contrast to the textbook example of 13‐ and 17‐year periodical cicadas. This interspecific variation tells us little about the underlying selective pressures, but still marks the intricate evolutionary pathways that have led to the observable diversity of developmental periods. A difficulty with developmental periods for over 99% of living organisms, which are ectotherms, is that simple durations are limitedly informative. Immature development in ectotherms is strongly influenced by environmental factors, most of all by temperature (Taylor 1981; Couret and Benedict 2014). Moreover, the strength of developmental response to temperature change may vary among comparison units (clones, populations, or species), that is, these may have different sensitivity to temperature (Gupta and Lewontin 1982; Parker 1984; Guntrip and Sibly 1998). This is why one dealing with ectotherms has to shift from rather ambiguous development time to the developmental norm of reaction to temperature.

### Thermal reaction norms for development: an overview

The relationship between development time and temperature can be described with a hyperbola‐like curve (Ratte 1984; Kipyatkov and Lopatina 2015). Unfortunately, these intuitively straightforward hyperbolic reaction norms are difficult to interpret and compare with each other, and so developmental rates (*R *=* *1/*D*) are more typically used. Rates of growth and development are also more relevant in terms of the underlying biochemical and biophysical machinery (de Jong and van der Have 2009). The reaction norm of developmental rate to temperature (which is further referred to simply as the thermal reaction norm, although biological processes other than development may have their own thermal reaction norms) is described by an asymmetrical bell‐shaped curve with a quasi‐linear portion in the nonstressful range of temperatures (Campbell et al. 1974; Ikemoto and Takai 2000).

Three modes of variation are proposed for nonlinear thermal reaction norms (Kingsolver et al. 2004; Izem and Kingsolver 2005; Knies et al. 2006): vertical shift (faster–slower), horizontal shift (hotter–colder), and generalist–specialist (broader–narrower). Although this model may work well with fitness‐related curves, its applicability in the analyses of growth and development rates seems to be limited. The distinction between vertical and horizontal shift is based on the stable or variable position of the point of “maximum performance at the optimal temperature” (Izem and Kingsolver 2005). However, the temperatures at which development is fastest are unnecessarily optimal for the functioning of the whole organism (Atkinson 1996; de Jong and van der Have 2009), and indeed, these temperatures often inflict significantly greater mortality (Lamb and Gerber 1985; Zahiri et al. 2010; Bahar et al. 2014). Furthermore, such severe constant heat is normally not experienced in nature (Campbell et al. 1974); for example, developmental optima in free‐living insects often exceed 30°C (Dillon and Frazier 2013). Hence, natural selection is unlikely to affect this thermal optimum directly, and the corresponding portion of the reaction norm is more of mechanistic than ecological or evolutionary interest. Similarly, ectotherms often avoid the season with very low constant temperatures by entering some form of dormancy. In the laboratory, these organisms may nonetheless develop at temperatures near the lower threshold, albeit extremely slowly. Experiments that make use of low‐temperature regimens yield characteristic concave‐up thermal reaction norms (Galkovskaja 1987; Jensen and Holmstrup 1997; Forster et al. 2011). The choice of the best model is thus undermined by the initial decision as to what part of the reaction norm is to be taken into account and what may be ignored.

Another approach focuses on the quasi‐linear portion of the reaction norm in the permissible temperature range (Campbell et al. 1974; Kipyatkov and Lopatina 2015). Comparisons of different models (Wagner et al. 1984; Kontodimas et al. 2004; Zahiri et al. 2010; Bahar et al. 2014) generally agree that the straight line is quite an accurate approximation over the mid‐temperature range. The linear regression equation is expressed as *R *= *a *+ *bT*, where *a* is the *y*‐intercept of the line. Two parameters are sufficient for a description of the line: the regression coefficient *b*, which is the measure of the slope, and the lower temperature threshold LTT = −*a*/*b*. The latter is the *x*‐intercept which is obtained by extrapolation of the line backwards to the point where *R *=* *0. Due to the nonlinearity of the whole reaction norm, it is more appropriate to regard the LTT as a base temperature above which the developmental rate will definitely be above zero. It is a biologically meaningful indicator of the position of the thermal reaction norm (especially of its lower part) relative to the temperature axis, so that populations and species with smaller LTT values are able to develop under colder conditions than those with greater LTT values. That being said, the LTT still overestimates the true threshold for development and, due to its extrapolated nature, may be subject to considerable inaccuracy (Campbell et al. 1974; but see below). In addition, linear reaction norms can be compared by elevation, which is calculated as a mean trait value across all regimens and shows the position of the line relative to the vertical axis (Zar 2010; Toftegaard et al. 2016).

It may seem that such truncation of the nonlinear reaction norm oversimplifies the problem. However, a more than 200‐year‐old practice of naturalists and agriculturalists shows that the linear model is congruent with the phenology of organisms in the field. As early as in the 18th century was it discovered that various crops had to accumulate a certain sum of temperatures for ripening and that this sum was the same in cool and hot years (Merriam 1894; Wang 1960). Later, this observation expanded into the concept of ectotherms' temperature‐independent physiological time (Taylor 1981; van Straalen 1983; Bonhomme 2000; Trudgill et al. 2005). Over recent decades, thousands of experimental studies have determined physiological time, which is usually referred to as the sum of degree‐days (SDD), and/or validated it under field conditions. The SDD can only be constant in relation to temperature when developmental rate increases with temperature linearly (van Straalen 1983). In fact, SDD = 1/*b*. This is why the researchers who estimate the SDD in the laboratory are so strongly concerned about the strict linearity of their data points and do not take into account the temperatures that are too high or too low (Ikemoto and Takai 2000; Nabity et al. 2006).

The overall good agreement between laboratory data (LTT and SDD) and field data (timing of phenological events) leads to two important conclusions: (1) the range of constant temperatures used to estimate LTT and SDD well approximates average environmental temperatures and (2) the corresponding part of the thermal reaction norm is the one most commonly expressed under natural conditions. Therefore, the linear portion of the thermal reaction norm is ecologically most relevant and from this follows its evolutionary relevance as well, because this portion should experience the strongest selective pressure. As a consequence, artificial selection on developmental rates within the linear range proves to be especially difficult (Neyfakh and Hartl 1993).

**Figure 1:**
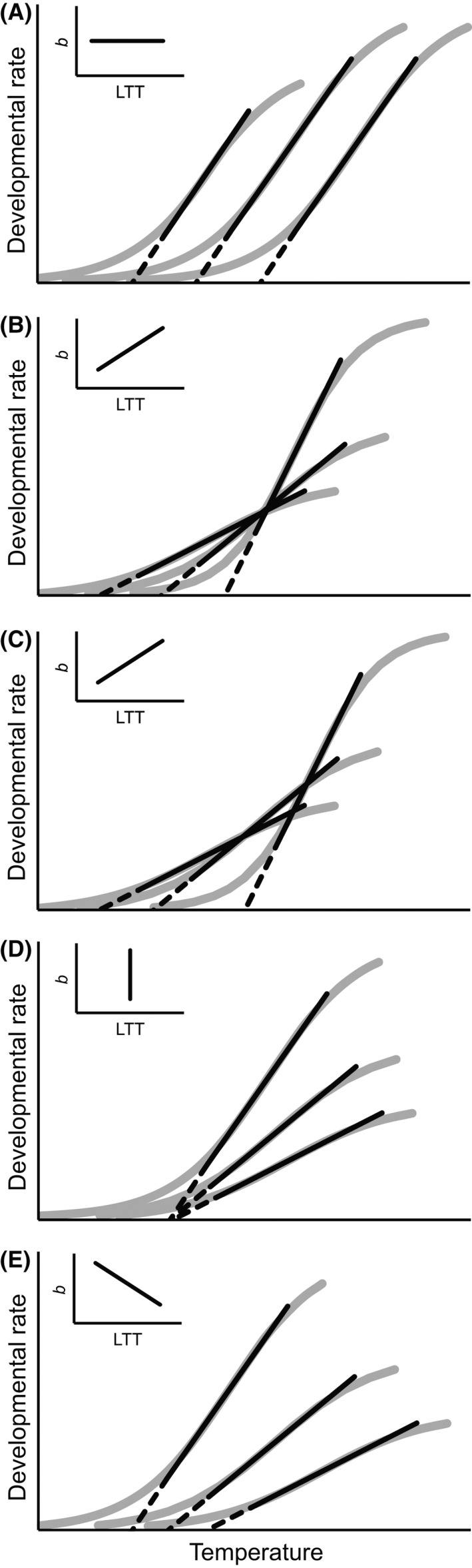
Possible patterns of variation of linear thermal reaction norms for development: (A) the slope is constant; (B) the slope and threshold are positively correlated, the intersection point is fixed; (C) the slope and threshold are positively correlated, the intersection point is floating; (D) the threshold is constant; (E) the slope and threshold are negatively correlated. Inset graphs show a respective relationship between the slope and lower temperature threshold.

Obrycki and Tauber (1982) were perhaps the first to recognize possible variation patterns of linear reaction norms for development. This classification in its final form (Honěk and Kocourek 1990) includes four patterns and bears some resemblance to the model proposed by Izem and Kingsolver (2005). The first variation pattern is parallel shift, which is characterized by the constant slope at different LTTs (Fig. 1A). The length of the linear portion may vary, but the vertical mode (“faster–slower”) and the horizontal mode (“hotter–colder”) are indistinguishable. The second pattern shows a positive correlation between the slope and LTT (Fig. 1B and C) and corresponds to the generalist–specialist trade‐off. In the third pattern, the LTT is constant, and all of the variation is created by different slopes (Fig. 1D). This so‐called isomorphic pattern is frequently found at the organismal level among developmental stages (Jarošík et al. 2004). The fourth type is characterized by a negative correlation between the slope and threshold (Fig. 1E). All of these outlined patterns may occur simultaneously in a large dataset.

### Positive slope–threshold correlation

The generalist–specialist trade‐off (Fig. 1B and C) seems to occur more frequently than the other patterns. It has often been discovered as a negative correlation between the SDD (1/*b*) and threshold (LTT). To avoid confusion, this widespread relationship is consistently referred to in this study as a positive correlation between the slope (*b*) and LTT. Also, the term “positive slope–threshold correlation” is preferable because it is purely descriptive and, unlike “generalist–specialist trade‐off”, does not a priori imply thermal adaptation or a physiological constraint.

Positive slope–threshold correlation is widely found both within and among populations (Tauber et al. 1987; Miller and LaMana 1995; Stacey and Fellowes 2002; Trudgill et al. 2005) and among species (Honěk and Kocourek 1990; Honěk 1996b; Li 1998; Ikemoto 2003; Bonato et al. 2011). In several cases, the interspecific variation seems to reflect the generalist–specialist trade‐off proper, because organisms as different as anurans (van der Have 2008), insects (Honěk 1996a; Kipyatkov and Lopatina 2015), nematodes, and plants (Trudgill et al. 2005) from higher latitudes have smaller thresholds and shallower slopes of developmental rate–temperature relationship than their counterparts from warmer climates (but see Irlich et al. 2009 for an alternative pattern in insects). Similarly, invasive species of insects have more temperature‐sensitive development than related noninvaders (Jarošík et al. 2015), and soil‐dwelling collembolans are more generalistic in relation to temperature than epigeic springtails (van Straalen 1994).

The positive slope–threshold correlation is often considered universal and even grounded in enzyme kinetics (Trudgill et al. 2005). As such, it is proposed to constrain the evolution of development time (Tauber et al. 1987; Stacey and Fellowes 2002); for example, selection for a lower LTT value would inadvertently decrease developmental rates at higher temperatures and the thermal sensitivity of development. This notion reaches its height in the “common‐intersection hypothesis” (Ikemoto 2003; Bonato et al. 2011), whereby interspecific differences in developmental rate arise from rotation of the reaction norm around a fixed point (as in Fig. 1B). However, intersection of thermal reaction norms in such a way implies that these are situated close to each other, and this scenario fails to explain the great differences in developmental rates that one observes in nature. According to a more plausible hypothesis (Honěk and Kocourek 1990), the positive slope–threshold correlation with a floating (not fixed) intersection point may be more pronounced at the level of populations and species. Further divergence eventually decouples the intersecting reaction norms, and comparisons of higher‐rank taxonomic groups would thus reveal a larger degree of parallel shift. In fact, one study (Kiritani 1991) shows that the positive slope–threshold correlation holds only within, and not among, insect groups.

Three issues undermine the biological universality of the positive slope–threshold correlation. First, the strength of this relationship within insect orders varies from substantial in Homoptera to negligible in Coleoptera and is accompanied by an enormous scatter of data (Honěk 1996b). Second, every multispecies dataset carries a genealogical structure which may dramatically confound the correlation between traits (Felsenstein 1985; Garland et al. 2005). Although Irlich et al. (2009) report very weak phylogenetic signal in insect rate–temperature relationships, the variance in the LTT and SDD is shown to increase at higher taxonomical levels (Jarošík et al. 2011), suggesting that thermal reaction norms do diverge. In the study of invasive versus noninvasive insect species (Jarošík et al. 2015), inclusion of taxonomical information in the analysis increases the positive slope–threshold correlation. This indeed should be so if the hypothesis of Honěk and Kocourek (1990) is correct. Third, from the mathematical point of view, the putative slope–threshold covariation can be an artifact of the positive correlation between *b* and −*a*, which is inherent in the linear model (*b *= [*R* − *a*]/*T*). Thereby, random variation around the regression line will always result in the positive relationship between the slope and threshold (Groeters 1992; Honěk 1996b; Irlich et al. 2009).

Thus, the ubiquity of the positive slope–threshold correlation in comparative studies of linear thermal reaction norms has two potential sources: the “true” generalist–specialist trade‐off, which likely does occur, and autocorrelation between *b* and LTT, which is produced by minor random differences and by poorly fit data. The former source can only be purified by minimizing the latter, that is, data should be meticulously selected and properly analyzed.

### “Warmer is better” principle

**Figure 2:**
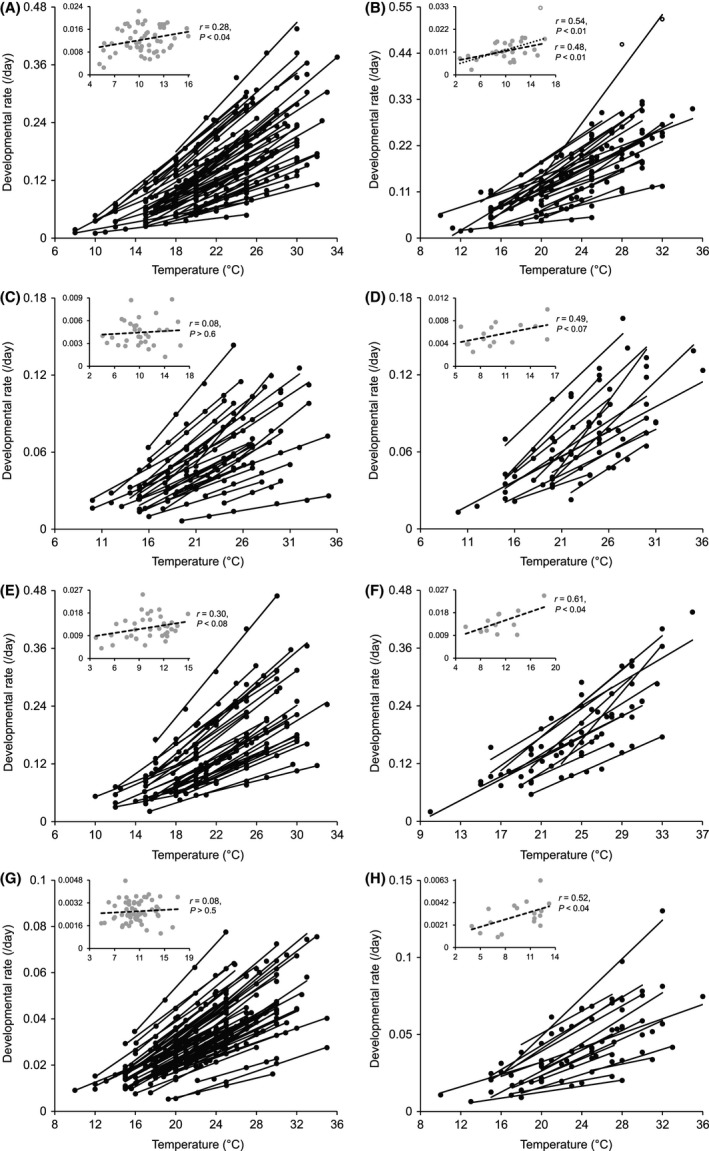
Thermal reaction norms for immature development in leaf beetles: A–B, eggs; C–D, larvae; E–F, pupae; G–H, total period to the adult stage. Left‐hand plates (A, C, E, and G) show “good” data where regression lines have an *r* ^2^ value no less than 0.980, and right‐hand plates (B, D, F, and H) show “bad” regression lines with *r* ^2^ < 0.980. Inset graphs illustrate a respective relationship between the slope and lower temperature threshold as in Figure 1. Open symbols denote an outlying regression line in the main graph of 2B and its parameters in the inset graph. Ordinary correlation analysis was repeated after including the outlier, which is shown by a dotted line in the inset graph.

Another controversy surrounds the extent to which the variation of thermal reaction norms is a product of thermodynamic constraints, namely the rate‐depressing effect of low temperatures (Clarke 2006; Angilletta et al. 2010). The problem starts with a distinction between two groups of species, cold‐adapted and warm‐adapted, which have reaction norms shifted to the colder and the hotter end of the temperature range, respectively (Clarke 1991, 2003; Frazier et al. 2006; de Jong and van der Have 2009). Species with right‐shifted thermal reaction norms (“warm‐adapted” ones) are proposed to outperform those whose thermal reaction norms are shifted to the left, even when both are compared under their own thermal optima (Frazier et al. 2006; Kingsolver and Huey 2008; Angilletta et al. 2010). Simply put, the record for the fastest development possible can only belong to a master of high temperatures. A master of low temperatures cannot be the quickest. Neither can a jack of all temperatures, because the generalist–specialist trade‐off (Fig. 1B and C) only aggravates the picture (Frazier et al. 2006). An opposite viewpoint (Clarke 2003) is that biological rates should be adaptively adjusted at a more or less the same level in all species at their respective “normal living temperatures,” regardless of the position of thermal reaction norms. In light of the above discussion of thermal optima, it must be stressed that Frazier et al. (2006) and Angilletta et al. (2010) misquote Clarke (2003), who does not infer the temperature optimum from maximal performance as they do (cf. figs. 1 and 2 in the former two papers, respectively, and box 1 in the latter one). Instead, he discusses concave‐up (Clarke 1991) and linear (Clarke 2003) dependences of physiological rates on temperature with “normal living temperatures” lying within the linear range (Clarke 2003). In terms of the linear model, the “warmer is better” principle may be rephrased like this: warmer is better, because species with greater LTT values tend to have more elevated thermal reaction norms than species with smaller LTTs.

## Aims of the Study

In order to test the outlined theoretical assumptions regarding developmental rate and its evolution, I have chosen the Chrysomelidae, a speciose family of insects that is extensively studied in terms of temperature‐dependent development. More specifically, I am asking: (1) What is the general pattern of the variation of linear thermal reaction norms for immature development in this family? (2) Is there a strong phylogenetic signal in the data and, if so, does it affect the variation pattern and in which way? (3) Does the improved quality of data weaken the positive correlation between the slope and *x*‐intercept (LTT)? (4) Is warmer better, that is, are the masters of high temperatures fastest? (5) Finally, which of the proposed factors actually constrain the evolution of development time?

## Materials and Methods

### Family under study

Leaf beetles (Chrysomelidae) are a promising group to approach the problem of the evolution of development time and thermal reaction norms from a macroecological perspective. This is one of the largest families with about 40,000 known species worldwide (Farrell and Sequeira 2004). While there is still some uncertainty about the basal phylogenetic relationships within the Chrysomelidae, the monophyly of most subfamilies is out of question (Reid 2014). The superfamily Chrysomeloidea which embraces leaf beetles, long‐horned beetles, and a few related groups is also well established to be monophyletic (Lawrence et al. 2011). Therefore, the interspecific diversity of any character in this group can be thought of as emanating from a single ancestral state. Many leaf beetles are agricultural or forest pests, whereas some other family members are used in the biological control of weeds, so their biology is relatively well studied.

### Data selection

I have been gathering all available studies on the temperature‐dependent development in the Chrysomelidae for 6 years in order to obtain as comprehensive a dataset as possible. I needed data that perfectly conformed to the linear relationship, which resulted in a number of strict criteria for the inclusion of experimental results in the following analysis. Some of these criteria agreed with previous recommendations (Shaffer 1983; Danks 2000) and/or were similar to those used by Irlich et al. (2009), whereas some others were developed empirically in the course of the work.

Primary data on the mean development time at each temperature had to be available. Studies reporting development time at one or two temperatures, or only regression equations, or only SDD and LTT were not taken into consideration. Data from graphs were used, where possible. Separate data on males and females were pooled together by calculating weighted means (if sample sizes were available) or simple means. Data on egg, larval, pupal, and total immature development were analyzed separately wherever possible. The prepupa was treated as part of the final larval instar, and in every case, it was ascertained that this transitional stage had not been combined with the pupa. Egg development time could not be quantified in some cases due to obligate embryonic diapause or viviparity, and so the larval + pupal period was considered as an equivalent of total development time. The inclusion of these species did not affect the results in any way. Studies with insufficiently detailed methods were cross‐checked with similar works on the same species to make sure that the reported data were reliable.

Temperatures should have been controlled at a constant level and accurately recorded. This was the main reason why works dating to 1950s and earlier were not considered.

There had to be at least three values of temperature per species. Series of experimental regimens often spanned beyond the permissible thermal range, and, when plotted against temperature, developmental rates showed a typical sigmoid pattern. In this case, the reaction norm was truncated to a linear region by excluding extreme values, and at least three temperatures should have remained.

Goodness of fit had to be sufficient. The exclusion criterion proposed by Ikemoto and Takai (2000) was not adopted because it only detected sigmoid deviations at higher and lower temperatures, thus providing an optimal thermal range within which the rate–temperature relationship was strictly linear. However, deviations from linearity often occurred at intermediate temperatures as well, for example, as a result of large interclutch variation, inaccurate measurement of temperature or errors in determining development time. Thus, all results were divided into two groups. “Good” data were satisfactorily fit by a straight line and had an *r* ^2^ not less than an empirically selected value of 0.980. The data that suffered from high variation around the line (*r* ^2 ^≤ 0.979) were not discarded but were labeled as “bad” and analyzed separately.

Each species was represented in the dataset only once. When the same species was studied by different authors and/or from different populations, data with a higher *r* ^2^ had priority. However, “good” and “bad” datasets were treated as independent samples, so their species lists partially overlapped. Some studies addressed interactive effects of temperature and diet or temperature and humidity. In such cases, survival rates were checked, and the regression line obtained under more favorable conditions was chosen. Development times recorded under short‐day conditions or including diapause were not used.

Mean development times were transformed into rates and regressed against temperature. Thus, the linear regression coefficient *b* and the LTT for each stage of each species were calculated anew and often did not coincide with previously reported values.

### Ordinary and phylogenetically informed correlation analyses

The correlation analyses focused on two pairs of traits. The prevailing variation pattern of linear reaction norms was determined from the relationship between the regression coefficient *b* and LTT (Fig. 1), and the evidence for “warmer is better” was sought by checking a positive relationship between the elevation of the reaction norm (i.e., mean developmental rate across all temperatures) and the LTT. For a start, I calculated ordinary Pearson's *r* in both cases, that is, assuming completely independent evolution of all species (Felsenstein 1985). All the ordinary statistical procedures, including those mentioned below, were carried out in STATISTICA 7.1 (StatSoft, Tulsa, OK, USA).

Phylogenetically informed analyses were performed on the same traits as follows. I built composite phylogenetic trees for the sets of “good” and “bad” data on egg, larval, pupal, and total immature development, relying on the best available phylogenies (Appendix S1). Branch lengths were initially set to unity (except for some internode branches that in case of a polytomy were set to zero) and then transformed according to the three methods available in the Editing module of PDTREE, namely the arbitrary branch lengths of Grafen, Pagel, and Nee (PDAP software: Garland et al. 1993 and references therein).

Trait correlations were tested using the Markov chain Monte Carlo (MCMC) procedure under the random walk model implemented in BayesTraits v2.0 software package (Meade and Pagel 2014). The MCMC technique can account for phylogenetic uncertainty by drawing each time a random tree from the collection of trees, so that the posterior distribution incorporates phylogenetic information from all the trees and is not based on any particular one (Pagel and Meade 2005). Thus, instead of choosing a better set of arbitrary branch lengths, I used a collection of four trees for each MCMC run. To test for a correlation between traits, the results of two MCMC runs were compared, one with the correlation coefficient searched by the Markov chain and the other one with the correlation set to zero (Meade and Pagel 2014). The residuals from the correlation were similarly tested for phylogenetic signal (Pagel's *λ*: Pagel 1997) by comparing the outcome of two MCMC runs, one in which *λ* was estimated and the other one in which *λ *= 0 (Meade and Pagel 2014). Two competing models were compared by calculating a log Bayes factor (log BF) which is double the difference between the harmonic mean of log‐marginal likelihood of the main model and that of the simpler model (Currie and Meade 2014). Evidence for the more complex model was considered as barely noteworthy when a log BF value was between 0 and 2, positive when the latter was between 2 and 6, strong when between 6 and 10, and very strong when over 10 (Kass and Raftery 1995).

Markov chains were allowed to produce a total of 1,010,000 iterations during each run. The optimal length of burn‐in (the period before convergence) was empirically estimated to be no more than 10,000 iterations. Of the remaining million, each 1000th value of log‐likelihood, correlation coefficient, and *λ* was sampled. Due to the probabilistic nature of Bayesian inference, each analysis was repeated three times to make sure that the outcome was consistent from run to run. Only the results of the first runs are reported because the subsequent trials did not reveal any significant inconsistency. As the Bayesian analyses returned posterior probability distributions (in contrast to common statistical methods that yielded a single value of parameter in interest), the results are expressed as medians with quartiles.

### Average intersection point of regression lines

All the regression equations were pairwise set equal to each other to determine the abscissas (i.e., temperature values) at which the respective lines crossed. The intersection abscissas were first averaged within each regression line separately; in this case, median values were calculated because the intersections were not normally distributed. These medians, one per line, had a normal distribution (Kolmogorov–Smirnov test, *P *>* *0.1), and so the average intersection point for a set of regression lines was expressed as an arithmetic mean. Also, in order to test the common‐intersection hypothesis, phylogenetic signal was measured in the samples of median intersection abscissas for eggs, larvae, pupae, and total development. Note that, in this case, the Pagel's *λ* was estimated for the trait (not for the error terms from any model), because the question was whether linear reaction norms for different species intersected at specific points, depending on the degree of relatedness. Phylogenetic signal was measured with BayesTraits v2.0 (Meade and Pagel 2014).

## Results

### The dataset

Appropriate developmental data were obtained from 122 published and unpublished sources, including personal communications and own experimental work. The final dataset (Appendix S2) includes 97 leaf beetle species studied at least during one developmental stage all over the globe from 1964 to 2015; seven relevant sources were discarded for various reasons, and a handful of difficultly accessible works still remains to be checked for the dataset to be exhaustive. The list of references is given in Appendix S3.

### Slope–threshold relationship and the influence of data quality

Overall, a positive slope–threshold relationship was found in all of the eight sets of regression lines, but its strength differed both among the developmental stages and between the sets of data with higher and lower goodness of linear fit. In the “good” data, the ordinary Pearson's correlation was weak and, except that for eggs, nonsignificant (Fig. 2A, C, E, and G). The “bad” data, when plotted, looked more irregular due to a larger amount of noise, and the slope–threshold correlation was strong and at least marginally significant, despite smaller sample sizes (Fig. 2B, D, F, and H).

In the “good” data, phylogenetic signal was generally strong both in the traits themselves and in the residuals from the correlations (Table 1), and large log BF values favored its inclusion in the models, except for the case of larval thermal reaction norms where the evidence for phylogenetic signal was weak. After correcting for phylogeny, the positive correlation between the slope and threshold became stronger and was well supported by log BF values (Table 2); only in larvae, this correlation remained weak and inconclusive. Traditional and phylogenetically informed methods thus provided somewhat conflicting evidence about the variation pattern of thermal reaction norms in leaf beetles. The former suggested parallel shift (Fig. 1A) as the predominant pattern, whereas the latter indicated a stronger positive relationship between the slope and LTT (Fig. 1B and C).

**Table 1:** Phylogenetic signal (Pagel's *λ*) in the parameters of thermal reaction norms and in the residuals from correlations between these parameters. The closer is *λ* to 1, the more similar are thermal phenotypes of related species. The results of Bayesian MCMC analyses are expressed as median values with lower and upper quartiles in brackets. Asterisks show the evidence for the presence of phylogenetic signal as compared with the model where *λ* is fixed at zero: *Log Bayes factor value between 2 and 6 (positive evidence); **Between 6 and 10 (strong evidence); ***>10 (very strong evidence). The absence of asterisk (log Bayes factor <2) means that *λ* is not significantly different from zero

Trait/correlation and data quality		Developmental stage			
		Eggs	Larvae	Pupae	Total development
Lower temperature threshold	Good data	0.64 (0.47–0.77)*	0.57 (0.39–0.77)*	0.42 (0.28–0.57)*	0.76 (0.64–0.84)***
	Bad data	0.33 (0.17–0.52)	0.41 (0.21–0.61)	0.40 (0.19–0.62)	0.23 (0.09–0.42)
Slope of the thermal reaction norm	Good data	0.63 (0.46–0.78)*	0.36 (0.19–0.55)	0.52 (0.34–0.70)*	0.50 (0.33–0.68)
	Bad data	0.28 (0.12–0.49)	0.54 (0.32–0.74)	0.38 (0.18–0.64)	0.45 (0.25–0.64)
Elevation of the thermal reaction norm	Good data	0.58 (0.43–0.72)**	0.42 (0.22–0.61)	0.56 (0.39–0.74)*	0.71 (0.54–0.85)*
Slope‐threshold correlation	Good data	0.75 (0.68–0.83)***	0.62 (0.44–0.77)	0.66 (0.51–0.78)***	0.84 (0.76–0.91)***
	Bad data	0.38 (0.21–0.56)	0.52 (0.33–0.71)	0.35 (0.16–0.58)	0.70 (0.56–0.81)**
Elevation‐threshold correlation	Good data	0.69 (0.58–0.77)***	0.56 (0.39–0.72)	0.58 (0.45–0.69)**	0.82 (0.74–0.90)***

**Table 2:** The results of phylogenetically informed correlation analyses. The correlation coefficients are expressed as median values with lower and upper quartiles in brackets. Asterisks correspond to log Bayes Factors that estimate the evidence for the given model, i.e., whether correlation is significantly different from zero: *Log Bayes factor value between 2 and 6 (positive evidence); **Between 6 and 10 (strong evidence); ***>10 (very strong evidence). The absence of asterisk (log Bayes factor <2) means that correlation is not significant

Correlation and data quality		Developmental stage			
		Eggs	Larvae	Pupae	Total development
Slope‐threshold correlation	Good data	0.46 (0.44–0.48)***	0.30 (0.25–0.36)*	0.55 (0.51–0.58)***	0.41 (0.38–0.43)**
	Bad data	0.57 (0.55–0.60)***	0.53 (0.49–0.57)*	0.61 (0.59–0.64)*	0.81 (0.71–0.84)***
Elevation‐threshold correlation	Good data	0.27 (0.25–0.29)*	0.11 (0.06–0.16)	0.36 (0.32–0.39)*	0.16 (0.14–0.18)

“Bad” regression lines contained less phylogenetic signal, as was expected due to their poorer fit. The Pagel's *λ* in the “bad” data was low and, except one case, not different from zero (Table 1), and so the outcome of MCMC runs for these data was largely congruent with the results of the ordinary correlation analysis (cf. correlation coefficients in Fig. 2B, D, F, H, and in Table 2). As high measurement uncertainty obliterated an important aspect of variation related to shared ancestry in the “bad” datasets, these were not further analyzed, and the following results solely refer to “good” data.

### Average intersection of reaction norms

**Figure 3:**
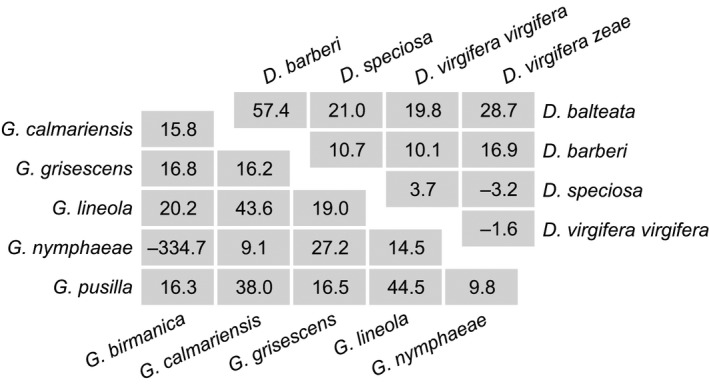
Abscissas (temperature values, °C) at which linear thermal reaction norms of different species in the genera *Galerucella* (total egg‐to‐adult development, bottom‐left) and *Diabrotica* (larval + pupal development, top‐right) intersect each other.

Three quarters of all intersection points lay outside the most commonly used experimental range from 15 to 30°C, which alone was incompatible with the common‐intersection hypothesis, whereby the common‐intersection temperature should be favorable for development. The mean across‐species intersection abscissas (11.8, 10.3, 12.2, and 11.9°C for eggs, larvae, pupae, and total development) were slightly higher than the corresponding mean LTTs for each developmental period (10.5, 10.2, 10.1, and 10.5°C, respectively). This could indicate a variation pattern similar to isomorphy (Fig. 1D) if the LTT values did not span such a wide range of approximately 12°C (Fig. 2). There were two especially well represented genera in the dataset (*Diabrotica* and *Galerucella*) which were remarkably illustrative of the absence of any regularity in the intersections of thermal reaction norms of closely related species (Fig. 3). There was practically no phylogenetic signal in the median intersection abscissas: the log BF values (model with *λ* estimated vs. model with *λ *= 0) for eggs, larvae, and pupae were negative and that for total development was too small (1.2) to be an important consideration.

### Elevation–threshold relationship

Ordinary correlation analyses showed an absence of any significant relationship between mean developmental rate (elevation of the reaction norm) and LTT. The Pearson's *r* ranged from 0.13 for pupae to −0.14 for total development (*P *>* *0.2). Phylogenetically informed analyses yielded positive evidence for “warmer is better” in eggs and pupae only and the correlation was weak (Table 2). Ultimately, in the case of total development, a negative log BF value indicated that the alternative model with the correlation fixed to zero was even favored.

## Discussion

### Inordinate variation of thermal reaction norms

This comparative study is based on a unique, large, and carefully collected dataset of temperature‐dependent parameters for immature development of leaf beetles. Thermal reaction norms for development are known for about a hundred leaf beetle species, which is exceptionally representative, compared with other families, even though this list comprises just 0.25% of the present‐day diversity of Chrysomelidae. Although species are not independent due to shared ancestry, studies carried out by different authors in remote parts of the globe are independent, even if they deal with closely related species. Strong phylogenetic signal (Table 1) therefore indicates that both the data and the trees compiled from numerous sources are reliable.

Given the high goodness‐of‐fit values for linear regression (*r* ^2^ > 0.979 in the “good” subset of data), I believe the estimates of the slope and threshold for each species to be quite accurate. However, by no means, these parameters of temperature‐dependent development may be treated as species‐specific constants. The real thermal reaction norm is neither a straight line nor even a curve. Many factors jointly affect the relationship between developmental rate and temperature, although their effects are often minor (Couret and Benedict 2014; Lopatina et al. 2014; Kutcherov et al. 2015). If one considers at least some of these variables, which may be discrete or continuous (e.g., sex, food quality, population density, photoperiod), the thermal reaction norm for developmental rate will turn into an intricate multidimensional body. A discussion of thermal reaction norms in terms of simple lines is the most radical reduction of this hardly imaginable complexity. Therefore, the linear reaction norms discussed here merely reflect some average temperature‐dependent development under more or less usual conditions. Even after such a reduction, the variation pattern of thermal reaction norms is rather intricate (Fig. 2), and the following sections will aim at disentangling this diversity.

### Data quality matters

The dataset presented here discriminates between the results with high and low measurement error, and so variation can be assessed separately in either subset (Fig. 2). The data with poorer linear fit contain less phylogenetic signal (Table 1), which is unsurprising, and show a variation pattern that is to be regarded as random or default (Groeters 1992; Irlich et al. 2009). It would be trivial to recommend using only the best available information when compiling a dataset. Less obvious is the fact that the inclusion of lower‐quality data always affects the resulting variation pattern in the same way, namely by increasing the positive slope–threshold correlation. Thus, caution should be exercised in calling any intersection of thermal reaction norms a generalist–specialist pattern as it may arise merely by chance.

### Related species have similar thermal parameters

The relationships between the slope, *x*‐intercept (lower temperature threshold), and elevation of thermal reaction norms show a strong phylogenetic signal (Table 1). This is especially true for egg, pupal, and total developmental rates, the thermal parameters of which appear to be similar in related species. Larvae tend to have more diverse thermal phenotypes, probably due to their different ecology. Defoliators, miners, borers, and root feeders are pooled together in this study, which might have weakened the phylogenetic signal. Even if it was so, these results support the statement of Jarošík et al. (2011) that the parameters of temperature‐dependent development of unstudied species can be approximately inferred from known examples in related taxa.

### Reaction norms waddle apart

Although the positive slope–threshold correlation is ultimately supported, regardless of the data quality (Table 2), the source of this correlation in the “bad” and “good” data is arguably different. The numerous previous works reporting the positive relationship between thermal parameters neither explicitly mention a prior strict censorship of data nor take phylogeny into account. “Good” data on leaf beetles, where the measurement error is minimized, show mostly weak and nonsignificant slope–threshold correlation and wide scatter in the threshold values (Fig. 2A, C, E, G). However, this correlation becomes significant after correcting for phylogeny, indicating that the pattern of variation is not the same at different taxonomical levels. The positive slope–threshold correlation is likelier to be found down the phylogenetic tree, that is, within groups of recently diverged species, whereas among these groups parallel shift predominates. Thus, thermal reaction norms tend to waddle away from each other and intersect at different points until the divergent evolution eventually parts them. Such a scenario of thermal reaction norm evolution, even though not termed as such, has already been envisaged by Honěk and Kocourek (1990) and is outlined above in the introduction. To the best of my knowledge, the present study provides the first comparative evidence confirming their long‐underappreciated idea (but see Kiritani 1991). This is also illustrative of how macroevolution may create variation patterns which are qualitatively different from the results of microevolution.

The fact that the positive slope–threshold correlation can eventually be overcome, given enough divergence time, sheds a doubt that this correlation seriously constrains the evolution of developmental rate as suggested by Tauber et al. (1987) and Stacey and Fellowes (2002). Furthermore, the prevalence of this correlation at lower taxonomical levels may be merely a remainder of the nonadaptive variation found within populations (Miller and LaMana 1995; Balashov and Kipyatkov 2008).

### Intersection may occur anywhere

The common‐intersection hypothesis is not supported on several grounds for the Chrysomelidae. First, the majority of intersection points lie outside the thermal range within which development usually takes place. Second, the thermal reaction norms of leaf beetles cross on average slightly above the temperature axis, but this is accompanied by too large scatter (Fig. 3) which prevents from drawing any generalizations. Third, the absence of phylogenetic signal in the median intersection abscissas suggests that there is not even a tendency for regression lines to intersect close to each other. However, the studied leaf beetle species achieve approximately the same developmental rate when each develops under its own optimal thermal conditions. This is the subject of the following section.

### Suum cuique is better

The immature development of leaf beetles is possible over a wide range of temperatures, and this is perhaps why they present an exception to the “warmer is better” principle, which is visualized in Fig. 2 and quantified in Table 2. These results provide support for the alternative concept of temperature compensation (Clarke 2003), whereby species inhabiting different thermal environments should maintain their biological rates at similar levels. Still, there is a slight positive correlation between the elevation of the reaction norms and their position relative to the *x*‐axis (i.e., *x*‐intercept, or LTT), which may indicate that this compensation is not perfect and the fundamental thermodynamic constraint has not been fully overcome (Clarke 2003). It remains to be tested whether right‐ and left‐shifted thermal reaction norms mirror adaptation to warm and cool climate, respectively. The scope of the study limits me to a sole remark that various factors other than environmental temperature may steadily set the pace of biological rates for consecutive generations (Dmitriew 2011; Kingsolver et al. 2012; Glazier 2015) and hence that of whole thermal reaction norms (Toftegaard et al. 2016).

## Conclusions

The questions raised in the introductory part receive the following answers. (1) Linear thermal reaction norms for immature development in the Chrysomelidae evolved primarily by parallel shift, which is reflected in the peculiar pattern of interspecific variation. In other words, selection for faster or slower development ultimately resulted in respectively faster or slower rates over the whole favorable thermal range, and the parameters of temperature‐dependent development could evolve more or less independently of each other. (2) The thermal reaction norms for immature development are similar in related species, which has important basic and practical implications. (3) Lower‐quality data exhibit a more happenstance variation pattern and weaker phylogenetic signal than better‐quality data do. This finding emphasizes the importance of scrupulous selection of developmental data prior to comparative analyses. (4) Warmer is not better; instead, mean developmental rates are similar in the studied leaf beetle species when each is tested in its own permissible temperature range. (5) In the case of leaf beetles, the progressing divergence of thermal reaction norms for development has largely overcome a number of proposed evolutionary constraints, albeit often imperfectly. Thus, the “tyranny” of enzyme thermodynamics over the life histories of ectotherms may not be as powerful as previously thought.

## Conflict of Interest

None declared.

## Supporting information


**Appendix S1** Composite phylogenetic trees used in comparative analyses.

**Appendix S2** Parameters of temperature‐dependent immature development in leaf beetles.

**Appendix S3** References for the dataset.
