# Supplementary material for: Thermal reaction norms can surmount evolutionary constraints: comparative evidence across leaf beetle species
Source: Ecol Evol. 2016 Jun 12;6(14):4670–83. doi: 10.1002/ece3.2231 (PMC4979698; doi:10.1002/ece3.2231)
Supplement: Supplementary file 1 — Appendix S1 Composite phylogenetic trees used in comparative analyses. [file ECE3-6-4670-s001.pdf]

Appendix 1

Composite phylogenetic trees used in comparative analyses

1.1. General remarks

Basal relationships among major lineages, subfamilies, and tribes were adopted from Gómez-Zurita et al. 2008. For further genealogy at lower taxonomical levels, I consulted phylogenetic studies of particular groups (subfamily Bruchinae: Kergoat et al. 2015; subfamily Cassidinae including Hispinae: Chaboo 2007; subfamily Criocerinae: Matsumura et al. 2014; subfamily Galerucinae including Alticinae: Gillespie et al. 2008; Ge et al. 2012; subtribe Diabroticina: Eben and Espinosa de los Monteros 2013; genus *Diabrotica*: Clark et al. 2001; genus *Galerucella*: Borghuis et al. 2009). All of these published trees were pruned to contain only relevant species.

The full species list and eight resultant synthetic topologies (with all non-zero branch lengths set to unity) are provided below. Trees in NEXUS format with different sets of arbitrary branch lengths are available from the author upon request.

“Good data” refer to regression lines of developmental rate on temperature with r^2 no less than 0.980, and “bad data” have an r^2 value of 0.979 or lower.

1.2. Species list

1. *Acalymma vittatum* (Fabricius, 1775)
2. *Acanthoscelides obtectus* (Say, 1831)
3. *Agasicles hygrophila* Selman et Vogt, 1971
4. *Agelastica alni* (Linnaeus, 1758)
5. *Altica carduorum* (Guérin-Méneville, 1858)
6. *Altica litigata* Fall, 1910
7. *Aphthona abdominalis* (Duftschmid, 1825)
8. *Aphthona cyparissiae* (Koch, 1803)
9. *Aphthona flava* (Guillebeau, 1895)
10. *Argopistes coccinelliformis* Csiki, 1940
11. *Bromius obscurus* (Linnaeus, 1758)
12. *Brontispa longissima* (Gestro, 1885)
13. *Bruchidius incarnatus* (Bohemann, 1833)
14. *Bruchus pisorum* (Linnaeus, 1758)
15. *Callosobruchus chinensis* (Linnaeus, 1758)
16. *Callosobruchus maculatus* (Fabricius, 1775)
17. *Callosobruchus rhodesianus* (Pic, 1902)
18. *Caryedon serratus* (Olivier, 1790)
19. *Cassida nebulosa* Linnaeus, 1758
20. *Cassida rubiginosa* Müller, 1776
21. *Cerotoma arcuata* (Olivier, 1791)
22. *Cerotoma ruficornis* (Olivier, 1791)
23. *Cerotoma trifurcata* (Forster, 1771)
24. *Chrysolina aeruginosa* (Faldermann, 1835)

25. *Chrysolina aurichalcea* (Gebler in Mannerheim, 1825)
26. *Chrysomela populi* (Linnaeus, 1758)
27. *Chrysomela scripta* Fabricius, 1801
28. *Chrysomela vigintipunctata* (Scopoli, 1763)
29. *Chrysophtharta agricola* (Chapuis, 1877)
30. *Colaphellus bowringi* Baly, 1865
31. *Crioceris asparagi* (Linnaeus, 1758)
32. *Crioceris quatuordecimpunctata* (Scopoli, 1763)
33. *Demotina fasciculata* (Baly, 1874)
34. *Diabrotica balteata* LeConte, 1865
35. *Diabrotica barberi* R. Smith et Lawrence, 1967
36. *Diabrotica speciosa* (Germar, 1824)
37. *Diabrotica virgifera virgifera* LeConte, 1868
38. *Diabrotica virgifera zeae* Krysan et Smith, 1980
39. *Diorhabda elongata* Brullé, 1832
40. *Diorhabda tarsalis* Weise, 1889
41. *Donacia provosti* Fairmaire, 1885
42. *Entomoscelis americana* Brown, 1942
43. *Eucolaspis puncticollis* (Broun, 1880)
44. *Galeruca circassica* Reitter, 1889
45. *Galeruca sardoa* (Gené, 1839)
46. *Galerucella birmanica* Jacoby, 1889
47. *Galerucella calmariensis* (Linnaeus, 1767)
48. *Galerucella grisescens* (Joannis, 1865)
49. *Galerucella lineola* (Fabricius, 1781)
50. *Galerucella nymphaeae* (Linnaeus, 1758)
51. *Galerucella pusilla* (Duftschmid, 1825)
52. *Gastrolina depressa* Baly, 1859
53. *Gastrophysa atrocyanea* Motschulsky, 1860
54. *Gastrophysa polygoni* (Linnaeus, 1758)
55. *Gastrophysa viridula* (De Geer, 1775)
56. *Gratiana boliviana* Spaeth, 1926
57. *Gratiana graminea* (Klug, 1829)
58. *Lema cyanella* Linnaeus, 1758
59. *Lema decempunctata* Gebler, 1830
60. *Lema diversa* Baly, 1873
61. *Lema scutellaris* (Kraatz, 1879)
62. *Leptinotarsa decemlineata* (Say, 1824)
63. *Lilioceris faldermanni* (Guerin-Meneville, 1829)
64. *Longitarsus bethae* Savini et Escalona, 2005
65. *Longitarsus flavicornis* (Stephens, 1831)
66. *Metriona elatior* (Klug, 1829)
67. *Microtheca ochroloma* Stål, 1860
68. *Monolepta hieroglyphica* (Motchulsky, 1858)
69. *Octodonta nipae* (Maulik, 1921)
70. *Ophraella communa* LeSage, 1986
71. *Oulema duftschmidi* (Redtenbacher, 1874)
72. *Oulema gallaeciana* (Heyden, 1870)
73. *Oulema melanopus* (Linnaeus, 1758)
74. *Oulema oryzae* (Kuwayama, 1931)
75. *Paropsis atomaria* Olivier, 1807
76. *Paropsis charybdis* Stål, 1860
77. *Phaedon brassicae* Baly, 1874
78. *Phratora vulgatissima* (Linnaeus, 1758)
79. *Phyllotreta armoraciae* (Koch, 1803)
80. *Phyllotreta cruciferae* (Goeze, 1777)
81. *Phyllotreta nemorum* (Linnaeus, 1758)
82. *Phyllotreta vittula* (Redtenbacher, 1949)
83. *Plagiodera versicolora* (Laicharting, 1781)
84. *Platyphora quadrisignata* (Germar,1824)
85. *Plesispa reichei* (Chapuis, 1875)
86. *Psylliodes chalcomerus* (Illiger 1807)
87. *Psylliodes chrysocephalus* (Linnaeus, 1758)
88. *Pyrrhalta viburni* (Paykull, 1799)
89. *Sclerophaedon orbicularis* (Suffrian, 1851)
90. *Systena basalis* DuVal, 1856
91. *Timarcha goettingensis normanna* Reiche, 1872
92. *Timarcha maritima* Perris, 1855
93. *Trachyaphthona nigrita* Ohno, 1961
94. *Trachyaphthona sordida* (Baly, 1874)
95. *Xanthogaleruca luteola* (Müller, 1766)
96. *Zygogramma bicolorata* Pallister, 1953
97. *Zygogramma suturalis* (Fabricius, 1775)

1.3. Trees

Eggs, good data

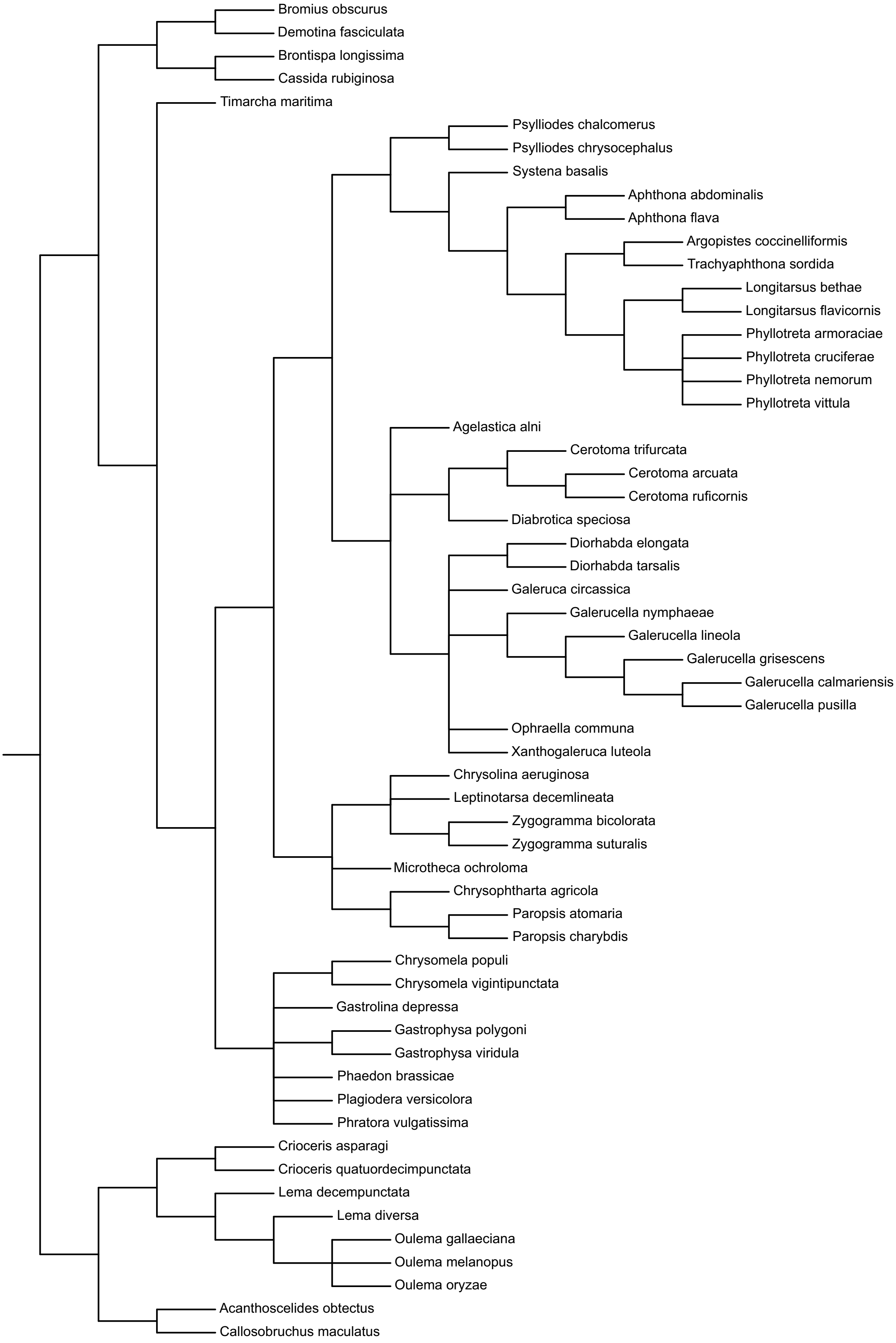

Eggs, bad data

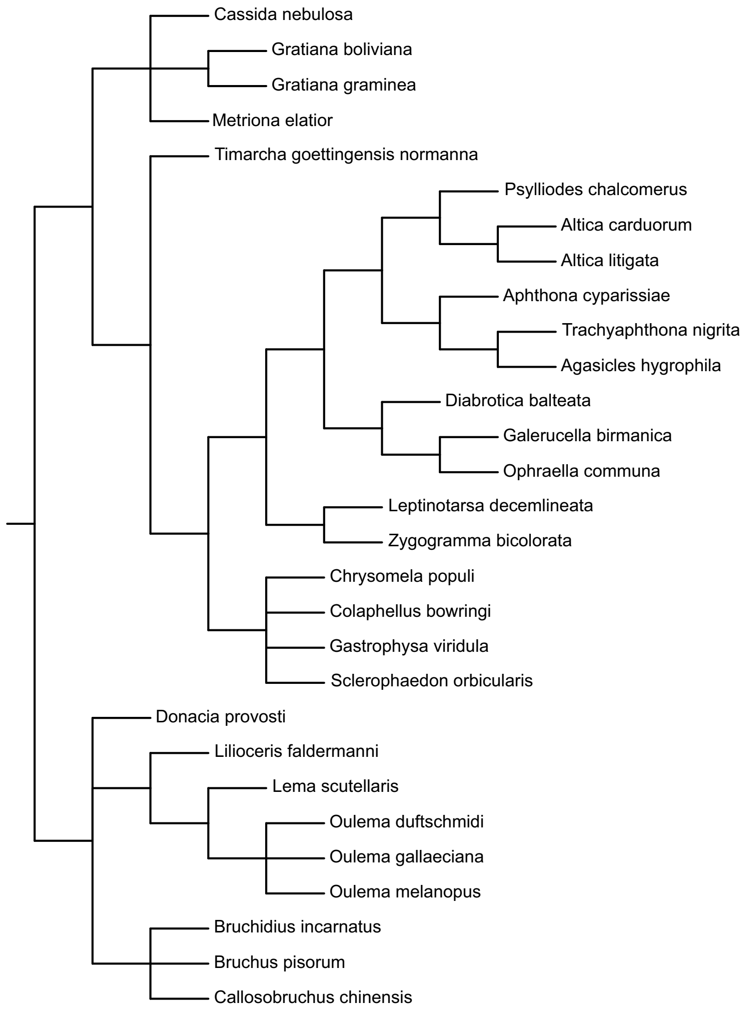

Larvae, good data

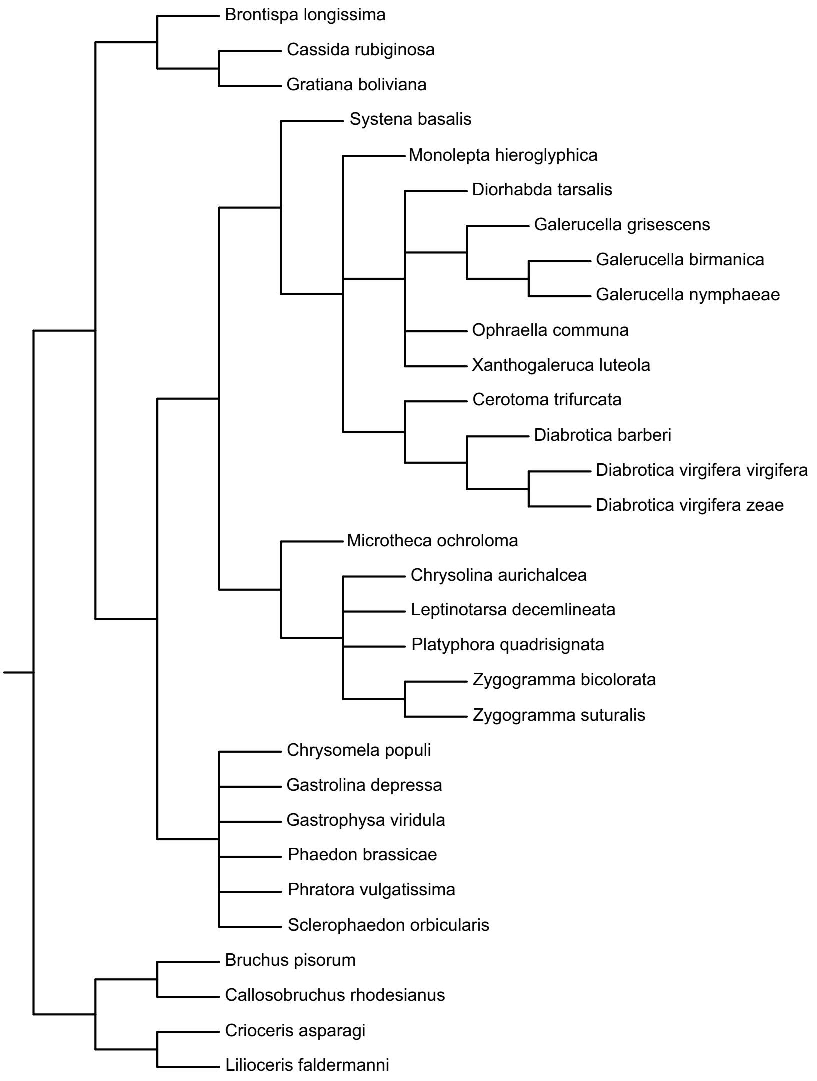

Larvae, bad data

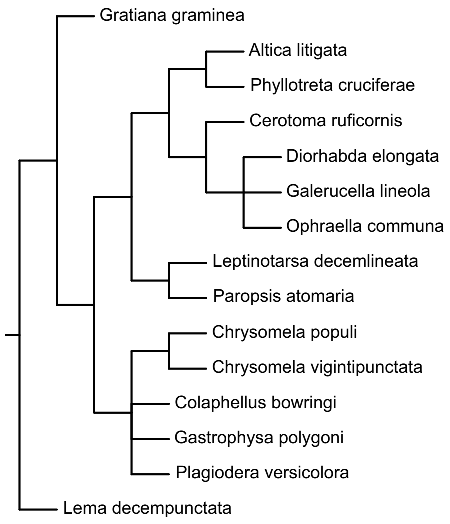

Pupae, good data

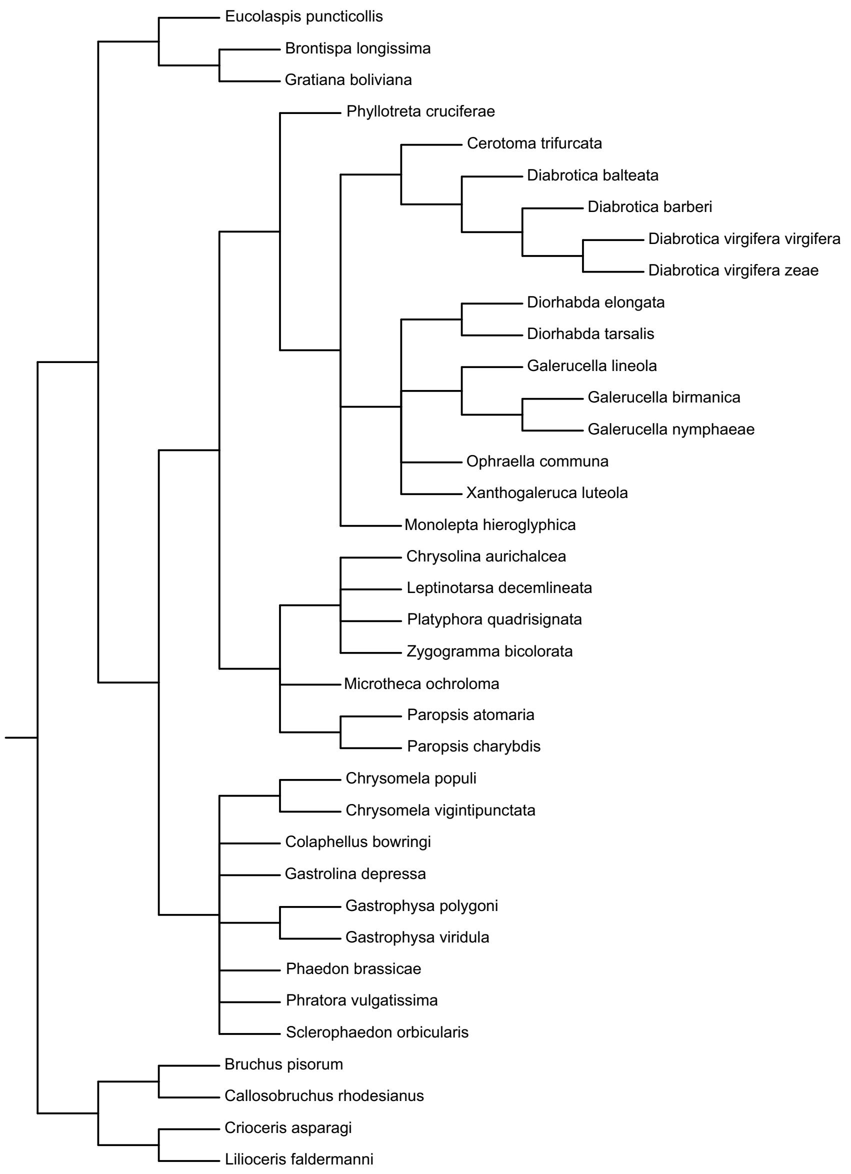

Pupae, bad data

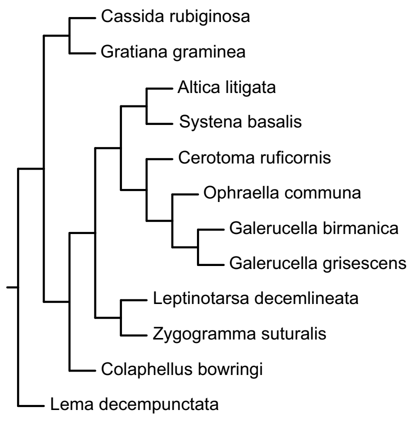

Total immature period, good data

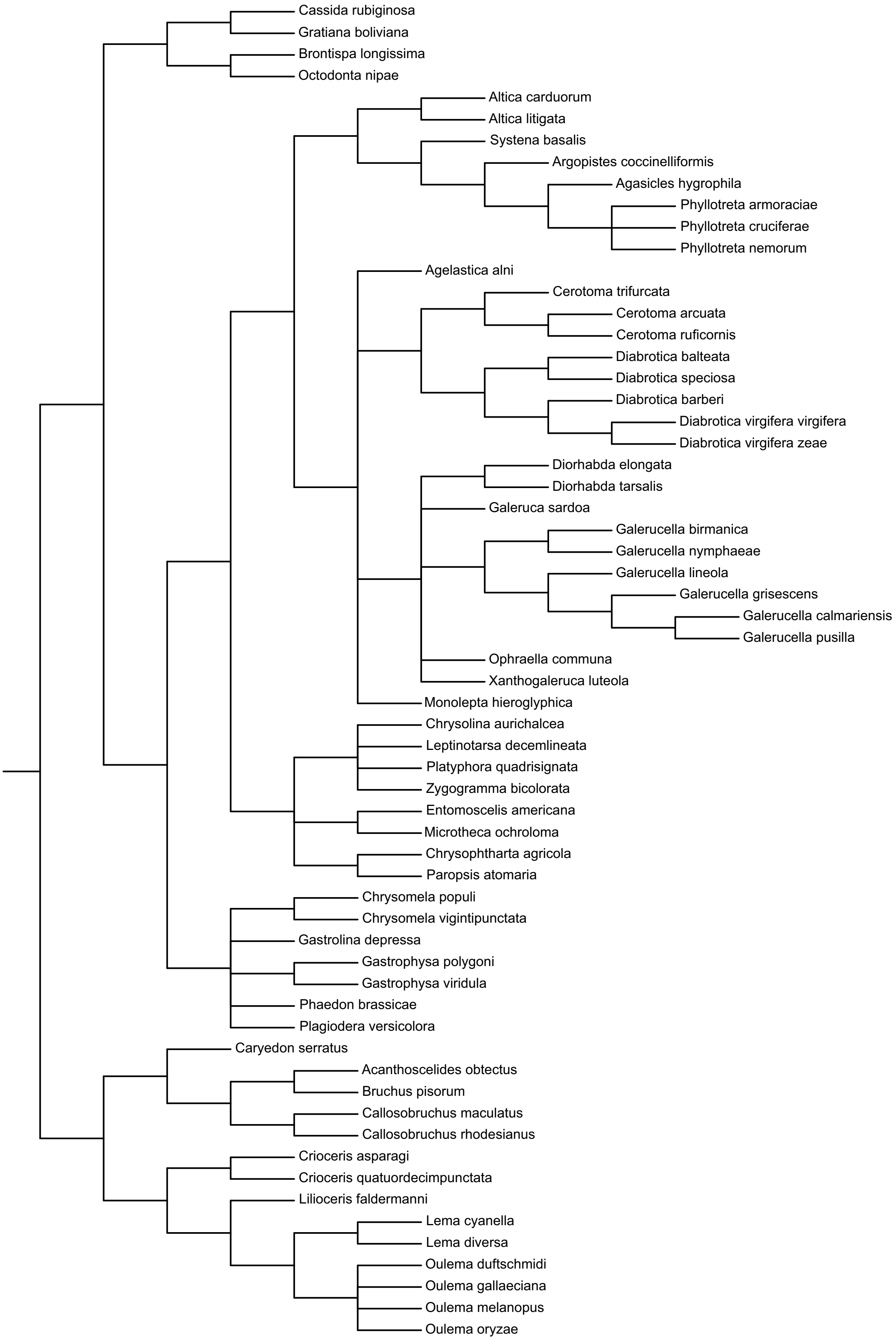

Total immature period, bad data

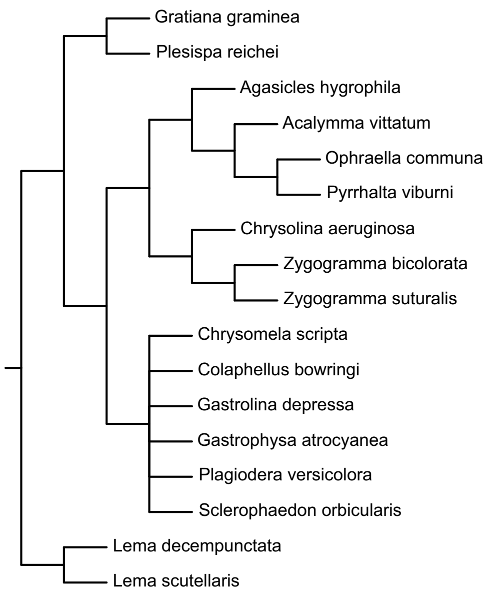

References

Borghuis, A., J. van Groenendael, O. Madsen, and J. Ouborg. 2009. Phylogenetic analyses of the leaf beetle genus Galerucella: evidence for host switching at speciation? Molecular Phylogenetics and Evolution 53:361–367.

Chaboo, C.S. 2007. Biology and phylogeny of the Cassidinae Gyllenhal sensu lato (tortoise and leaf-mining beetles) (Coleoptera: Chrysomelidae). Bulletin of the American Museum of Natural History 305:1–250.

Clark, T. L., L. J. Meinke, and J. E. Foster. 2001. Molecular phylogeny of Diabrotica beetles (Coleoptera: Chrysomelidae) inferred from analysis of combined mitochondrial and nuclear DNA sequences. Insect Molecular Biology 10:303–314.

Eben, A. and A. Espinosa de los Monteros. 2013. Tempo and mode of evolutionary radiation in Diabroticina beetles (genera Acalymma, Cerotoma, and Diabrotica). ZooKeys 332: 207–321.

Ge, D., J. Gómez-Zurita, D. Chesters, X. Yang, and A. P. Vogler. 2012. Suprageneric systematics of flea beetles (Chrysomelidae: Alticinae) inferred from multilocus sequence data. Molecular Phylogenetics and Evolution 62:793–805.

Gillespie, J. J., D. W. Tallamy, E. G. Riley, and A. I. Cognato. 2008. Molecular phylogeny of rootworms and related galerucine beetles (Coleoptera: Chrysomelidae). Zoologica Scripta 37:195–222.

Gómez-Zurita, J., T. Hunt, and A. P. Vogler. 2008. Multilocus ribosomal RNA phylogeny of the leaf beetles (Chrysomelidae). Cladistics 24:34–50.

Kergoat, G. J., B. P. Le Ru, S. E. Sadeghi, M. Tuda, C. A. M. Reid, Z. György, G. Genson, C. S. Ribeiro-Costa, and A. Delobel. 2015. Evolution of Spermophagus seed beetles (Coleoptera, Bruchinae, Amblycerini) indicates both synchronous and delayed colonizations of host plants. Molecular Phylogenetics and Evolution 89:91–103.

Matsumura, Y., I. Yao, R. G. Beutel, and K. Yoshizawa. 2014. Molecular phylogeny of the leaf beetle subfamily Criocerinae (Coleoptera: Chrysomelidae) and the correlated evolution of reproductive organs. Arthropod Systematics and Phylogeny 72:95–110.
