# Supplementary material for: Thermal reaction norms can surmount evolutionary constraints: comparative evidence across leaf beetle species
Source: Ecol Evol. 2016 Jun 12;6(14):4670–83. doi: 10.1002/ece3.2231 (PMC4979698; doi:10.1002/ece3.2231)
Supplement: Supplementary file 2 — Appendix S2 Parameters of temperature‐dependent immature development in leaf beetles. [file ECE3-6-4670-s002.docx]

**Appendix 2
Parameters of temperature-dependent immature development in leaf beetles**

The following dataset is based on an extensive survey of literature and own experimental work. The relationship between developmental rate (*R*) and temperature (*T*) was approximated with a linear regression equation *R* = *a* + *bT*, which is justifiable in the non-stressful temperature range within which development normally takes place. The tables contain the values of the regression coefficient, or the slope of the rate-temperature relationship (*b*), y-intecept (*a*), x-intercept (lower temperature threshold -*a*/*b*), sum of degree-days (1/*b*), and elevation (mean developmental rate across all the experimental temperatures used). Data that had high goodness-of-fit (*r*^2^≥0.980) were considered reliable (“good”) and may be safely used in comparative analyses. “Bad” data (*r*^2^<0.980) may serve as guide values, but should be treated with caution if precision is needed.

Species with embryonic diapause or viviparity are denoted with an asterisk. In these cases, total development was measured from hatching/birth to adult emergence (i.e., excluding the egg stage).

**Eggs, good data**

| **Species** | **Sub- family** | **Regression coefficient** | **Intercept** | **Lower temperature threshold** | **Sum of degree-days** | **Elevation** | **r^2^** | **Data source** |
| --- | --- | --- | --- | --- | --- | --- | --- | --- |
| *Acanthoscelides obtectus* (Say, 1831) | Bruch | **0.0117** | -0.1477 | **12.62** | 85.48 | 0.1155 | **0.994** | Howe, Currie, 1964 |
| *Callosobruchus maculatus* (Fabricius, 1775) | Bruch | **0.0163** | -0.2582 | **15.81** | 61.23 | 0.1583 | **0.990** | Gong et al., 2000 |
| *Brontispa longissima* (Gestro, 1885) | Cass | **0.0165** | -0.2155 | **13.03** | 60.45 | 0.1981 | **0.996** | Giang, Nakamura, 2009 |
| *Cassida rubiginosa* Müller, 1776 | Cass | **0.0119** | -0.1456 | **12.23** | 83.98 | 0.1461 | **0.998** | Ward, Pienkowski, 1978 |
| *Chrysolina aeruginosa* (Faldermann, 1835) | Chrys | **0.0129** | -0.1841 | **14.33** | 77.82 | 0.1114 | **0.985** | Wei et al., 2013 |
| *Chrysomela populi* (Linnaeus, 1758) | Chrys | **0.0151** | -0.1438 | **9.54** | 66.33 | 0.1867 | **0.998** | Kutcherov et al., 2011 |
| *Chrysomela vigintipunctata* (Scopoli, 1763) | Chrys | **0.0182** | -0.1797 | **9.88** | 54.98 | 0.2019 | **0.980** | Kutcherov, 2015 |
| *Chrysophtharta agricola* Chapuis, 1877 | Chrys | **0.0180** | -0.1542 | **8.57** | 55.54 | 0.1771 | **0.986** | Nahrung et al., 2004 |
| *Gastrolina depressa* Baly, 1859 | Chrys | **0.0179** | -0.1483 | **8.30** | 55.99 | 0.2155 | **0.997** | Kutcherov, 2016 |
| *Gastrophysa polygoni* (Linnaeus, 1758) | Chrys | **0.0178** | -0.1858 | **10.45** | 56.22 | 0.2589 | **0.999** | Xi et al., 2000 |
| *Gastrophysa viridula* (De Geer, 1775) | Chrys | **0.0170** | -0.1497 | **8.82** | 58.91 | 0.2052 | **0.996** | Kucherov, Kipyatkov, 2011 |
| *Leptinotarsa decemlineata* (Say, 1824) | Chrys | **0.0146** | -0.1589 | **10.90** | 68.58 | 0.1702 | **0.997** | Boukal et al., 2015 |
| *Microtheca ochroloma* Stål, 1860 | Chrys | **0.0112** | -0.1081 | **9.66** | 89.34 | 0.1437 | **0.994** | Manrique et al., 2012 |
| *Paropsis atomaria* Olivier, 1807 | Chrys | **0.0094** | -0.0958 | **10.24** | 106.89 | 0.0913 | **0.999** | Nahrung et al., 2008 |
| *Paropsis charybdis* Stål, 1860 | Chrys | **0.0162** | -0.1192 | **7.36** | 61.80 | 0.1613 | **0.994** | McGregor, 1989 |
| *Phaedon brassicae* Baly, 1874 | Chrys | **0.0110** | -0.0735 | **6.67** | 90.70 | 0.1249 | **0.991** | Wang et al., 2007 |
| *Phratora vulgatissima* (Linnaeus, 1758) | Chrys | **0.0086** | -0.0487 | **5.65** | 116.10 | 0.1078 | **0.990** | Pollard, 2014 |
| *Plagiodera versicolora* (Laicharting, 1781) | Chrys | **0.0188** | -0.1846 | **9.84** | 53.31 | 0.2531 | **0.997** | Yang et al., 2006 |
| *Timarcha maritima* Perris, 1855 | Chrys | **0.0025** | -0.0144 | **5.68** | 394.16 | 0.0276 | **0.992** | Chevin, 1992 |
| *Zygogramma bicolorata* Pallister, 1953 | Chrys | **0.0112** | -0.0957 | **8.52** | 89.05 | 0.1712 | **0.992** | King, 2008 |
| *Zygogramma suturalis* (Fabricius, 1775) | Chrys | **0.0177** | -0.2216 | **12.50** | 56.40 | 0.1330 | **0.999** | Igrc, 1989 |
| *Crioceris asparagi* (Linnaeus, 1758) | Crioc | **0.0155** | -0.1232 | **7.94** | 64.43 | 0.1695 | **0.982** | Taylor, Harcourt, 1978 |
| *Crioceris quatuordecimpunctata* (Scopoli, 1763) | Crioc | **0.0224** | -0.2228 | **9.96** | 44.72 | 0.3139 | **0.984** | Chiba, Shinohe, 1975 |
| *Lema decempunctata* Gebler, 1830 | Crioc | **0.0149** | -0.1647 | **11.04** | 67.03 | 0.2232 | **0.986** | Du et al., 2006 |
| *Lema diversa* Baly, 1873 | Crioc | **0.0177** | -0.2265 | **12.78** | 56.43 | 0.2431 | **0.983** | Cheng et al., 2001 |
| *Oulema gallaeciana* (Heyden, 1870) | Crioc | **0.0127** | -0.1312 | **10.30** | 78.54 | 0.1235 | **0.998** | Ali et al., 1977 |
| *Oulema melanopus* (Linnaeus, 1758) | Crioc | **0.0114** | -0.1144 | **10.01** | 87.53 | 0.1141 | **0.997** | Ali et al., 1977 |
| *Oulema oryzae* (Kuwayama, 1931) | Crioc | **0.0190** | -0.2043 | **10.74** | 52.56 | 0.1763 | **1.000** | Syoji, 1972 |
| *Bromius obscurus* (Linnaeus, 1758) | Eumol | **0.0104** | -0.1170 | **11.21** | 95.83 | 0.1114 | **0.997** | Kutcherov et al., 2014 |
| *Demotina fasciculata* (Baly, 1874) | Eumol | **0.0065** | -0.0666 | **10.27** | 154.30 | 0.0792 | **0.997** | Yoshizaki, Ozawa, 2009 |
| *Agelastica alni* (Linnaeus, 1758) | Galer | **0.0098** | -0.0958 | **9.74** | 101.66 | 0.1102 | **0.997** | Kutcherov et al., 2014 |
| *Aphthona abdominalis* (Duftschmid, 1825) | Galer | **0.0136** | -0.2173 | **16.00** | 73.66 | 0.1221 | **0.998** | Fornasari, 1995 |
| *Aphthona flava* (Guillebeau, 1895) | Galer | **0.0058** | -0.0616 | **10.69** | 173.43 | 0.0571 | **0.996** | Maw, 1981 |
| *Argopistes coccinelliformis* Csiki, 1940 | Galer | **0.0086** | -0.0872 | **10.20** | 116.88 | 0.1053 | **0.995** | Tamura, Takeuchi, 1992 |
| *Cerotoma arcuata* (Olivier, 1791) | Galer | **0.0095** | -0.1305 | **13.74** | 105.31 | 0.1069 | **0.987** | Nava, Parra, 2003 |
| *Cerotoma ruficornis* (Olivier, 1791) | Galer | **0.0083** | -0.1076 | **12.98** | 120.72 | 0.1047 | **0.980** | Heyer et al., 1988 |
| *Cerotoma trifurcata* (Forster, 1771) | Galer | **0.0103** | -0.1439 | **13.93** | 96.82 | 0.1014 | **0.996** | Zeiss et al., 1996 |
| *Diabrotica speciosa* (Germar, 1824) | Galer | **0.0084** | -0.0946 | **11.22** | 118.58 | 0.1120 | **0.985** | Milanez, Parra, 2000 |
| *Diorhabda elongata* Brullé, 1832 | Galer | **0.0097** | -0.0950 | **9.79** | 103.07 | 0.1476 | **0.997** | Herrera et al., 2005 |
| *Diorhabda tarsalis* Weise, 1889 | Galer | **0.0078** | -0.0918 | **11.83** | 128.88 | 0.0944 | **0.995** | Zhang et al., 2007 |
| *Galeruca circassica* Reitter, 1889 | Galer | **0.0111** | -0.0636 | **5.74** | 90.31 | 0.1578 | **1.000** | Aslan et al., 2005 |
| *Galerucella calmariensis* (Linnaeus, 1767) | Galer | **0.0095** | -0.0851 | **8.93** | 104.89 | 0.1055 | **1.000** | McAvoy, Kok, 2004 |
| *Galerucella grisescens* (Joannis, 1866) | Galer | **0.0137** | -0.1465 | **10.69** | 72.97 | 0.1961 | **0.999** | Lin et al., 2002 |
| *Galerucella lineola* (Fabricius, 1781) | Galer | **0.0103** | -0.0873 | **8.51** | 97.48 | 0.0974 | **0.990** | Pollard, 2014 |
| *Galerucella nymphaeae* (Linnaeus, 1758) | Galer | **0.0129** | -0.1153 | **8.94** | 77.59 | 0.1569 | **0.998** | Tauber et al., 1996 |
| *Galerucella pusilla* (Duftschmid, 1825) | Galer | **0.0100** | -0.1034 | **10.32** | 99.79 | 0.0970 | **0.999** | McAvoy, Kok, 2004 |
| *Longitarsus bethae* Savini et Escalona, 2005 | Galer | **0.0055** | -0.0621 | **11.31** | 182.27 | 0.0724 | **0.991** | Simelane, 2007 |
| *Longitarsus flavicornis* (Stephens, 1831) | Galer | **0.0038** | -0.0195 | **5.17** | 264.66 | 0.0419 | **0.992** | Ireson et al., 1991 |
| *Ophraella communa* LeSage, 1986 | Galer | **0.0184** | -0.2472 | **13.45** | 54.43 | 0.1368 | **0.999** | Emura, 1999 |
| *Phyllotreta armoraciae* (Koch, 1803) | Galer | **0.0167** | -0.2095 | **12.58** | 60.03 | 0.1736 | **1.000** | Vig, 2003 |
| *Phyllotreta cruciferae* (Goeze, 1777) | Galer | **0.0133** | -0.1588 | **11.96** | 75.32 | 0.1466 | **1.000** | Vig, 2003 |
| *Phyllotreta nemorum* (Linnaeus, 1758) | Galer | **0.0167** | -0.2109 | **12.62** | 59.83 | 0.1736 | **1.000** | Vig, 2003 |
| *Phyllotreta vittula* (Redtenbacher, 1949) | Galer | **0.0177** | -0.2317 | **13.07** | 56.40 | 0.1761 | **0.999** | Vig, 2003 |
| *Psylliodes chalcomerus* (Illiger, 1807) | Galer | **0.0076** | -0.0543 | **7.12** | 131.04 | 0.0983 | **0.996** | Cristofaro et al., 2003 |
| *Psylliodes chrysocephalus* (Linnaeus, 1758) | Galer | **0.0062** | -0.0396 | **6.45** | 162.59 | 0.0588 | **1.000** | Mathiasen et al., 2015 |
| *Systena basalis* DuVal, 1856 | Galer | **0.0065** | -0.0793 | **12.28** | 154.90 | 0.0670 | **0.988** | Chiang Lok et al., 1987 |
| *Trachyaphthona sordida* (Baly, 1874) | Galer | **0.0082** | -0.0996 | **12.14** | 121.87 | 0.0836 | **0.992** | Okamoto et al., 2008 |
| *Xanthogaleruca luteola* (Müller, 1766) | Galer | **0.0140** | -0.1675 | **11.94** | 71.28 | 0.1440 | **0.999** | King et al., 1985 |

**Eggs, bad data**

| **Species** | **Sub- family** | **Regression coefficient** | **Intercept** | **Lower temperature threshold** | **Sum of degree-days** | **r^2^** | **Data source** |
| --- | --- | --- | --- | --- | --- | --- | --- |
| *Bruchidius incarnatus* (Bohemann, 1833) | Bruch | **0.0094** | -0.1421 | **15.11** | 106.35 | **0.973** | El-Kifl, Metwally, 1971 |
| *Bruchus pisorum* (Linnaeus, 1758) | Bruch | **0.0145** | -0.1557 | **10.75** | 69.02 | **0.933** | Smith, 1992 |
| *Callosobruchus chinensis* (Linnaeus, 1758) | Bruch | **0.0173** | -0.2818 | **16.26** | 57.72 | **0.954** | Deng et al., 1999 |
| *Cassida nebulosa* Linnaeus, 1758 | Cass | **0.0105** | -0.1379 | **13.09** | 94.98 | **0.956** | Redžepagić et al., 1983 |
| *Gratiana boliviana* Spaeth, 1926 | Cass | **0.0127** | -0.1424 | **11.25** | 79.03 | **0.950** | Diaz et al., 2008 |
| *Gratiana graminea* (Klug, 1829) | Cass | **0.0122** | -0.1364 | **11.18** | 81.97 | **0.921** | Manrique et al., 2012 |
| *Metriona elatior* (Klug, 1829) | Cass | **0.0090** | -0.0879 | **9.75** | 111.02 | **0.979** | Gandolfo et al., 2008 |
| *Chrysomela populi* (Linnaeus, 1758) | Chrys | **0.0154** | -0.1517 | **9.88** | 65.11 | **0.970** | Gomi et al., 2005 |
| *Colaphellus bowringi* Baly, 1865 | Chrys | **0.0164** | -0.1816 | **11.07** | 60.97 | **0.969** | Xue et al., 2002 |
| *Gastrophysa viridula* (De Geer, 1775) | Chrys | **0.0154** | -0.1284 | **8.34** | 64.98 | **0.971** | Honěk et al., 2003 |
| *Leptinotarsa decemlineata* (Say, 1824) | Chrys | **0.0178** | -0.2336 | **13.11** | 56.14 | **0.976** | Lactin, Holliday, 1992 |
| *Sclerophaedon orbicularis* (Suffrian, 1851) | Chrys | **0.0079** | -0.0214 | **2.70** | 126.14 | **0.972** | Fischer, 1985 |
| *Timarcha goettingensis normanna* Reiche, 1872 | Chrys | **0.0024** | -0.0111 | **4.54** | 408.18 | **0.967** | Chevin, 1991 |
| *Zygogramma bicolorata* Pallister, 1953 | Chrys | **0.0092** | -0.0367 | **4.00** | 109.09 | **0.955** | Hasan, Shafiq Ansari, 2015 |
| *Lema scutellaris* (Kraatz, 1879) | Crioc | **0.0324** | -0.5043 | **15.57** | 30.89 | **0.875** | Zhang et al., 1995 |
| *Lilioceris faldermanni* (Guerin-Meneville, 1829) | Crioc | **0.0161** | -0.1388 | **8.64** | 62.28 | **0.915** | Mojib et al., 2013 |
| *Oulema duftschmidi* (Redtenbacher, 1874) | Crioc | **0.0162** | -0.2015 | **12.47** | 61.89 | **0.970** | Morlacchi et al., 2007 |
| *Oulema gallaeciana* (Heyden, 1870) | Crioc | **0.0113** | -0.0958 | **8.50** | 88.74 | **0.976** | Walczak, 2005 |
| *Oulema melanopus* (Linnaeus, 1758) | Crioc | **0.0106** | -0.0837 | **7.87** | 94.03 | **0.979** | Walczak, 2005 |
| *Donacia provosti* Fairmaire, 1885 | Donac | **0.0059** | -0.0638 | **10.72** | 168.16 | **0.940** | Qin et al., 2009 |
| *Agasicles hygrophila* Selman et Vogt, 1971 | Galer | **0.0180** | -0.2276 | **12.67** | 55.67 | **0.974** | Stewart et al., 1999 |
| *Altica carduorum* (Guérin-Méneville, 1858) | Galer | **0.0101** | -0.0854 | **8.50** | 99.50 | **0.954** | Wan et al., 1996 |
| *Altica litigata* Fall, 1910 | Galer | **0.0112** | -0.1064 | **9.47** | 89.04 | **0.969** | Pettis, Braman, 2007 |
| *Aphthona cyparissiae* (Koch, 1803) | Galer | **0.0062** | -0.0700 | **11.36** | 162.27 | **0.951** | Maw, 1981 |
| *Diabrotica balteata* LeConte, 1865 | Galer | **0.0122** | -0.1777 | **14.61** | 82.19 | **0.960** | Heyer, Cruz, 1983 |
| *Galerucella birmanica* Jacoby, 1889 | Galer | **0.0117** | -0.1161 | **9.94** | 85.65 | **0.964** | Zheng et al., 2008 |
| *Ophraella communa* LeSage, 1986 | Galer | **0.0110** | -0.1220 | **11.10** | 91.01 | **0.807** | Zhou Z.-S. et al., 2010 |
| *Psylliodes chalcomerus* (Illiger 1807) | Galer | **0.0072** | -0.0385 | **5.33** | 138.46 | **0.977** | Cristofaro et al., 2003 |
| *Trachyaphthona nigrita* Ohno, 1961 | Galer | **0.0072** | -0.0814 | **11.26** | 138.32 | **0.892** | Okamoto et al., 2008 |

**Larvae, good data**

| **Species** | **Sub- family** | **Regression coefficient** | **Intercept** | **Lower temperature threshold** | **Sum of degree-days** | **Elevation** | **r^2^** | **Data source** |
| --- | --- | --- | --- | --- | --- | --- | --- | --- |
| *Bruchus pisorum* (Linnaeus, 1758) | Bruch | **0.0012** | -0.0174 | **14.10** | 809.94 | 0.0164 | **0.996** | Smith, Ward, 1995 |
| *Callosobruchus rhodesianus* (Pic, 1902) | Bruch | **0.0048** | -0.0689 | **14.40** | 209.01 | 0.0447 | **0.993** | Howe, Currie, 1964 |
| *Brontispa longissima* (Gestro, 1885) | Cass | **0.0022** | -0.0256 | **11.46** | 447.60 | 0.0235 | **0.999** | Zhong et al., 2005 |
| *Cassida rubiginosa* Müller, 1776 | Cass | **0.0042** | -0.0466 | **11.02** | 236.66 | 0.0457 | **1.000** | Ward, Pienkowski, 1978 |
| *Gratiana boliviana* Spaeth, 1926 | Cass | **0.0059** | -0.0947 | **16.16** | 170.78 | 0.0761 | **0.997** | Diaz et al., 2008 |
| *Chrysolina aurichalcea* (Gebler in Mannerheim, 1825) | Chrys | **0.0028** | -0.0182 | **6.60** | 361.84 | 0.0313 | **1.000** | Fujiyama, Harada, 1996 |
| *Chrysomela populi* (Linnaeus, 1758) | Chrys | **0.0056** | -0.0478 | **8.55** | 178.79 | 0.0786 | **0.989** | Gomi et al., 2005 |
| *Gastrolina depressa* Baly, 1859 | Chrys | **0.0087** | -0.0754 | **8.66** | 114.82 | 0.1024 | **0.999** | Kutcherov, 2016 |
| *Gastrophysa viridula* (De Geer, 1775) | Chrys | **0.0062** | -0.0445 | **7.16** | 161.04 | 0.0857 | **0.997** | Kucherov, Kipyatkov, 2011 |
| *Leptinotarsa decemlineata* (Say, 1824) | Chrys | **0.0039** | -0.0384 | **9.85** | 256.41 | 0.0435 | **0.999** | Boukal et al., 2015 |
| *Microtheca ochroloma* Stål, 1860 | Chrys | **0.0065** | -0.0640 | **9.91** | 154.81 | 0.0652 | **0.999** | Manrique et al., 2012 |
| *Phaedon brassicae* Baly, 1874 | Chrys | **0.0060** | -0.0457 | **7.56** | 165.52 | 0.0631 | **0.998** | Wang et al., 2007 |
| *Phratora vulgatissima* (Linnaeus, 1758) | Chrys | **0.0031** | -0.0148 | **4.81** | 325.96 | 0.0410 | **0.991** | Pollard, 2014 |
| *Platyphora quadrisignata* (Germar,1824) | Chrys | **0.0034** | -0.0268 | **7.93** | 295.57 | 0.0408 | **0.996** | Schroder et al., 1994 |
| *Sclerophaedon orbicularis* (Suffrian, 1851) | Chrys | **0.0040** | -0.0164 | **4.10** | 249.82 | 0.0436 | **0.990** | Fischer, 1985 |
| *Zygogramma bicolorata* Pallister, 1953 | Chrys | **0.0027** | -0.0206 | **7.74** | 376.47 | 0.0543 | **0.999** | Hasan, Shafiq Ansari, 2015 |
| *Zygogramma suturalis* (Fabricius, 1775) | Chrys | **0.0048** | -0.0629 | **13.17** | 209.34 | 0.0442 | **0.991** | Igrc, 1989 |
| *Crioceris asparagi* (Linnaeus, 1758) | Crioc | **0.0054** | -0.0509 | **9.49** | 186.30 | 0.0761 | **0.992** | Taylor, Harcourt, 1978 |
| *Lilioceris faldermanni* (Guerin-Meneville, 1829) | Crioc | **0.0038** | -0.0212 | **5.62** | 265.05 | 0.0543 | **0.993** | Mojib et al., 2013 |
| *Cerotoma trifurcata* (Forster, 1771) | Galer | **0.0031** | -0.0321 | **10.24** | 318.55 | 0.0393 | **0.999** | McCreary, 2013 |
| *Diabrotica barberi* R. Smith et Lawrence, 1967 | Galer | **0.0027** | -0.0269 | **9.82** | 364.76 | 0.0307 | **0.997** | Woodson, Jackson, 1988 |
| *Diabrotica virgifera virgifera* LeConte, 1868 | Galer | **0.0038** | -0.0388 | **10.14** | 261.34 | 0.0415 | **0.979** | Jackson, Elliott, 1988 |
| *Diabrotica virgifera zeae* Krysan et Smith, 1980 | Galer | **0.0032** | -0.0353 | **10.95** | 309.82 | 0.0331 | **0.997** | Woodson, Chandler, 2000 |
| *Diorhabda tarsalis* Weise, 1889 | Galer | **0.0048** | -0.0446 | **9.25** | 207.44 | 0.0711 | **0.987** | Zhang et al., 2007 |
| *Galerucella birmanica* Jacoby, 1889 | Galer | **0.0048** | -0.0483 | **10.02** | 207.51 | 0.0602 | **0.996** | Chen, Chen, 2003 |
| *Galerucella grisescens* (Joannis, 1866) | Galer | **0.0088** | -0.1340 | **15.21** | 113.51 | 0.0863 | **0.990** | Lin et al., 2002 |
| *Galerucella nymphaeae* (Linnaeus, 1758) | Galer | **0.0053** | -0.0486 | **9.16** | 188.39 | 0.0561 | **0.996** | Tauber et al., 1996 |
| *Monolepta hieroglyphica* (Motchulsky, 1858) | Galer | **0.0027** | -0.0335 | **12.29** | 366.38 | 0.0388 | **0.993** | Li et al., 2008 |
| *Ophraella communa* LeSage, 1986 | Galer | **0.0070** | -0.0887 | **12.65** | 142.58 | 0.0708 | **0.999** | Emura, 1999 |
| *Systena basalis* DuVal, 1856 | Galer | **0.0027** | -0.0451 | **16.55** | 367.09 | 0.0298 | **0.987** | Chiang Lok et al., 1987 |
| *Xanthogaleruca luteola* (Müller, 1766) | Galer | **0.0039** | -0.0444 | **11.42** | 257.01 | 0.0419 | **1.000** | King et al., 1985 |

**Larvae, bad data**

| **Species** | **Sub- family** | **Regression coefficient** | **Intercept** | **Lower temperature threshold** | **Sum of degree-days** | **r^2^** | **Data source** |
| --- | --- | --- | --- | --- | --- | --- | --- |
| *Gratiana graminea* Klug, 1829 | Cass | **0.0042** | -0.0408 | **9.61** | 235.53 | **0.891** | Manrique et al., 2012 |
| *Chrysomela populi* (Linnaeus, 1758) | Chrys | **0.0072** | -0.0929 | **12.82** | 137.97 | **0.975** | Kutcherov et al., 2011 |
| *Chrysomela vigintipunctata* (Scopoli, 1763) | Chrys | **0.0057** | -0.0481 | **8.39** | 174.36 | **0.943** | Kutcherov, 2015 |
| *Colaphellus bowringi* Baly, 1865 | Chrys | **0.0078** | -0.0762 | **9.83** | 128.91 | **0.959** | Dong et al., 2007 |
| *Gastrophysa polygoni* (Linnaeus, 1758) | Chrys | **0.0070** | -0.0394 | **5.66** | 143.70 | **0.931** | Hilterhaus, 1965 |
| *Leptinotarsa decemlineata* (Say, 1824) | Chrys | **0.0035** | -0.0324 | **9.15** | 282.01 | **0.968** | Zhou Z.-X. et al., 2010 |
| *Paropsis atomaria* Olivier, 1807 | Chrys | **0.0025** | -0.0179 | **7.10** | 395.89 | **0.971** | Nahrung et al., 2008 |
| *Plagiodera versicolora* (Laicharting, 1781) | Chrys | **0.0068** | -0.0637 | **9.31** | 146.20 | **0.959** | Yang et al., 2006 |
| *Lema decempunctata* Gebler, 1830 | Crioc | **0.0039** | -0.0255 | **6.54** | 256.99 | **0.940** | Du et al., 2006 |
| *Altica litigata* Fall, 1910 | Galer | **0.0048** | -0.0399 | **8.37** | 209.59 | **0.953** | Pettis, Braman, 2007 |
| *Cerotoma ruficornis* (Olivier, 1791) | Galer | **0.0047** | -0.0756 | **16.00** | 211.62 | **0.922** | Heyer et al., 1988 |
| *Diorhabda elongata* Brullé, 1832 | Galer | **0.0070** | -0.1026 | **14.61** | 142.38 | **0.975** | Herrera et al., 2005 |
| *Galerucella lineola* (Fabricius, 1781) | Galer | **0.0039** | -0.0248 | **6.40** | 257.83 | **0.970** | Pollard, 2014 |
| *Ophraella communa* LeSage, 1986 | Galer | **0.0100** | -0.1604 | **16.04** | 99.97 | **0.910** | Zhou et al., 2010 |
| *Phyllotreta cruciferae* (Goeze, 1777) | Galer | **0.0042** | -0.0499 | **11.83** | 237.20 | **0.974** | Kinoshita et al., 1979 |

**Pupae, good data**

| **Species** | **Sub- family** | **Regression coefficient** | **Intercept** | **Lower temperature threshold** | **Sum of degree-days** | **Elevation** | **r^2^** | **Data source** |
| --- | --- | --- | --- | --- | --- | --- | --- | --- |
| *Bruchus pisorum* (Linnaeus, 1758) | Bruch | **0.0067** | -0.0823 | **12.22** | 148.48 | 0.0650 | **0.994** | Smith, Ward, 1995 |
| *Callosobruchus rhodesianus* (Pic, 1902) | Bruch | **0.0128** | -0.1587 | **12.45** | 78.42 | 0.1441 | **0.996** | Howe, Currie, 1964 |
| *Brontispa longissima* (Gestro, 1885) | Cass | **0.0138** | -0.1662 | **12.03** | 72.41 | 0.1100 | **0.994** | Zhong et al., 2005 |
| *Gratiana boliviana* Spaeth, 1926 | Cass | **0.0129** | -0.1756 | **13.67** | 77.82 | 0.1713 | **0.984** | Diaz et al., 2008 |
| *Chrysolina aurichalcea* (Gebler in Mannerheim, 1825) | Chrys | **0.0084** | -0.0630 | **7.50** | 119.04 | 0.1011 | **1.000** | Fujiyama, Harada, 1996 |
| *Chrysomela populi* (Linnaeus, 1758) | Chrys | **0.0193** | -0.2216 | **11.46** | 51.71 | 0.2039 | **0.998** | Kutcherov et al., 2011 |
| *Chrysomela vigintipunctata* (Scopoli, 1763) | Chrys | **0.0194** | -0.1804 | **9.32** | 51.65 | 0.2271 | **0.996** | Kutcherov, 2015 |
| *Colaphellus bowringi* Baly, 1865 | Chrys | **0.0153** | -0.1443 | **9.45** | 65.46 | 0.2122 | **0.983** | Xue et al., 2002 |
| *Gastrolina depressa* Baly, 1859 | Chrys | **0.0253** | -0.2389 | **9.45** | 39.54 | 0.3171 | **0.997** | Kutcherov, 2016 |
| *Gastrophysa polygoni* (Linnaeus, 1758) | Chrys | **0.0153** | -0.1583 | **10.33** | 65.27 | 0.1481 | **0.997** | Hilterhaus, 1965 |
| *Gastrophysa viridula* (De Geer, 1775) | Chrys | **0.0136** | -0.0847 | **6.22** | 73.40 | 0.2304 | **0.998** | Honěk et al., 2003 |
| *Leptinotarsa decemlineata* (Say, 1824) | Chrys | **0.0115** | -0.1186 | **10.36** | 87.33 | 0.1387 | **0.998** | Boukal et al., 2015 |
| *Microtheca ochroloma* Stål, 1860 | Chrys | **0.0138** | -0.1167 | **8.46** | 72.47 | 0.1593 | **0.999** | Manrique et al., 2012 |
| *Paropsis atomaria* Olivier, 1807 | Chrys | **0.0078** | -0.0678 | **8.64** | 127.57 | 0.1027 | **0.999** | Nahrung et al., 2008 |
| *Paropsis charybdis* Stål, 1860 | Chrys | **0.0090** | -0.0724 | **8.04** | 111.05 | 0.0946 | **0.989** | McGregor, 1989 |
| *Phaedon brassicae* Baly, 1874 | Chrys | **0.0149** | -0.1091 | **7.32** | 67.09 | 0.1890 | **0.991** | Wang et al., 2007 |
| *Phratora vulgatissima* (Linnaeus, 1758) | Chrys | **0.0092** | -0.0515 | **5.61** | 108.83 | 0.1138 | **0.997** | Pollard, 2014 |
| *Platyphora quadrisignata* (Germar,1824) | Chrys | **0.0077** | -0.0668 | **8.66** | 129.56 | 0.0875 | **0.991** | Schroder et al., 1994 |
| *Sclerophaedon orbicularis* (Suffrian, 1851) | Chrys | **0.0082** | -0.0307 | **3.74** | 121.54 | 0.0875 | **0.995** | Fischer, 1985 |
| *Zygogramma bicolorata* Pallister, 1953 | Chrys | **0.0052** | -0.0507 | **9.73** | 191.91 | 0.0890 | **0.992** | Hasan, Shafiq Ansari, 2015 |
| *Crioceris asparagi* (Linnaeus, 1758) | Crioc | **0.0142** | -0.1844 | **12.98** | 70.40 | 0.1565 | **0.986** | Taylor, Harcourt, 1978 |
| *Lilioceris faldermanni* (Guerin-Meneville, 1829) | Crioc | **0.0052** | -0.0300 | **5.77** | 192.44 | 0.0635 | **0.999** | Mojib et al., 2013 |
| *Eucolaspis puncticollis* (Broun, 1880) | Eumol | **0.0040** | -0.0178 | **4.41** | 247.65 | 0.0428 | **0.994** | Doddala et al., 2013 |
| *Cerotoma trifurcata* (Forster, 1771) | Galer | **0.0103** | -0.1276 | **12.44** | 97.45 | 0.1060 | **0.994** | McCreary, 2013 |
| *Diabrotica balteata* LeConte, 1865 | Galer | **0.0118** | -0.1350 | **11.46** | 84.88 | 0.1351 | **0.987** | Heyer, Cruz, 1983 |
| *Diabrotica barberi* R. Smith et Lawrence, 1967 | Galer | **0.0096** | -0.1172 | **12.20** | 104.15 | 0.1133 | **0.998** | Woodson, Jackson, 1988 |
| *Diabrotica virgifera virgifera* LeConte, 1868 | Galer | **0.0090** | -0.0932 | **10.32** | 110.77 | 0.1099 | **0.996** | Jackson, Elliott, 1988 |
| *Diabrotica virgifera zeae* Krysan et Smith, 1980 | Galer | **0.0115** | -0.1495 | **12.96** | 86.67 | 0.1130 | **0.997** | Woodson, Chandler, 2000 |
| *Diorhabda elongata* Brullé, 1832 | Galer | **0.0109** | -0.1465 | **13.43** | 91.68 | 0.1262 | **1.000** | Herrera et al., 2005 |
| *Diorhabda tarsalis* Weise, 1889 | Galer | **0.0176** | -0.2636 | **14.97** | 56.78 | 0.1591 | **0.993** | Zhang et al., 2007 |
| *Galerucella birmanica* Jacoby, 1889 | Galer | **0.0177** | -0.1780 | **10.05** | 56.43 | 0.2384 | **0.987** | Zheng et al., 2008 |
| *Galerucella lineola* (Fabricius, 1781) | Galer | **0.0114** | -0.0975 | **8.56** | 87.77 | 0.1503 | **0.996** | Pollard, 2014 |
| *Galerucella nymphaeae* (Linnaeus, 1758) | Galer | **0.0187** | -0.1956 | **10.46** | 53.47 | 0.2252 | **0.999** | Tauber et al., 1996 |
| *Monolepta hieroglyphica* (Motchulsky, 1858) | Galer | **0.0088** | -0.1093 | **12.42** | 113.67 | 0.1107 | **0.989** | Li et al., 2008 |
| *Ophraella communa* LeSage, 1986 | Galer | **0.0165** | -0.1924 | **11.63** | 60.45 | 0.1840 | **0.999** | Emura, 1999 |
| *Phyllotreta cruciferae* (Goeze, 1777) | Galer | **0.0090** | -0.1061 | **11.83** | 111.43 | 0.1182 | **0.982** | Kinoshita et al., 1979 |
| *Xanthogaleruca luteola* (Müller, 1766) | Galer | **0.0129** | -0.1569 | **12.17** | 77.55 | 0.1293 | **0.996** | King et al., 1985 |

**Pupae, bad data**

| **Species** | **Sub- family** | **Regression coefficient** | **Intercept** | **Lower temperature threshold** | **Sum of degree-days** | **r^2^** | **Data source** |
| --- | --- | --- | --- | --- | --- | --- | --- |
| *Cassida rubiginosa* Müller, 1776 | Cass | **0.0131** | -0.1363 | **10.37** | 76.13 | **0.976** | Ward, Pienkowski, 1978 |
| *Gratiana graminea* Klug, 1829 | Cass | **0.0188** | -0.2632 | **14.02** | 53.27 | **0.860** | Manrique et al., 2012 |
| *Colaphellus bowringi* Baly, 1865 | Chrys | **0.0148** | -0.1357 | **9.17** | 67.58 | **0.952** | Hu et al., 2008 |
| *Leptinotarsa decemlineata* (Say, 1824) | Chrys | **0.0109** | -0.0979 | **8.97** | 91.67 | **0.974** | Logan et al., 1985 |
| *Zygogramma suturalis* (Fabricius, 1775) | Chrys | **0.0132** | -0.1761 | **13.34** | 75.72 | **0.954** | Igrc, 1989 |
| *Lema decempunctata* Gebler, 1830 | Crioc | **0.0124** | -0.0695 | **5.61** | 80.70 | **0.817** | Du et al., 2006 |
| *Altica litigata* Fall, 1910 | Galer | **0.0105** | -0.0842 | **8.06** | 95.67 | **0.979** | Pettis, Braman, 2007 |
| *Cerotoma ruficornis* (Olivier, 1791) | Galer | **0.0247** | -0.4487 | **18.16** | 40.47 | **0.895** | Heyer et al., 1988 |
| *Galerucella birmanica* Jacoby, 1889 | Galer | **0.0173** | -0.1883 | **10.87** | 57.69 | **0.968** | Chen, Chen, 2003 |
| *Galerucella grisescens* (Joannis, 1866) | Galer | **0.0175** | -0.1922 | **10.98** | 57.11 | **0.944** | Lin et al., 2002 |
| *Ophraella communa* LeSage, 1986 | Galer | **0.0094** | -0.1001 | **10.67** | 106.62 | **0.955** | Zhou Z.-S. et al., 2010 |
| *Systena basalis* DuVal, 1856 | Galer | **0.0092** | -0.1278 | **13.86** | 108.39 | **0.973** | Chiang Lok et al., 1987 |

**Total immature development, good data**

| **Species** | **Sub- family** | **Regression coefficient** | **Intercept** | **Lower temperature threshold** | **Sum of degree-days** | **Elevation** | **r^2^** | **Data source** |
| --- | --- | --- | --- | --- | --- | --- | --- | --- |
| *Acanthoscelides obtectus* (Say, 1831) | Bruch | **0.0028** | -0.0389 | **14.00** | 360.31 | 0.0278 | **0.992** | Kutcherov, unpublished |
| *Bruchus pisorum* (Linnaeus, 1758) | Bruch | **0.0011** | -0.0152 | **14.38** | 947.91 | 0.0102 | **0.999** | Smith, 1992; Smith, Ward, 1995 |
| *Callosobruchus maculatus* (Fabricius, 1775) | Bruch | **0.0036** | -0.0618 | **17.10** | 276.76 | 0.0358 | **1.000** | Gong et al., 2000 |
| *Callosobruchus rhodesianus* (Pic, 1902) | Bruch | **0.0027** | -0.0355 | **12.99** | 365.71 | 0.0294 | **0.996** | Howe, Currie, 1964 |
| *Caryedon serratus* (Olivier, 1790) | Bruch | **0.0015** | -0.0241 | **16.29** | 674.77 | 0.0166 | **0.999** | Mishra et al., 2012 |
| *Brontispa longissima* (Gestro, 1885) | Cass | **0.0017** | -0.0189 | **11.34** | 598.83 | 0.0178 | **0.999** | Zhong et al., 2005 |
| *Cassida rubiginosa* Müller, 1776 | Cass | **0.0023** | -0.0242 | **10.41** | 430.64 | 0.0327 | **0.996** | Ward, Pienkowski, 1978 |
| *Gratiana boliviana* Spaeth, 1926 | Cass | **0.0029** | -0.0402 | **13.73** | 341.31 | 0.0389 | **0.989** | Diaz et al., 2008 |
| *Octodonta nipae* (Maulik, 1921) | Cass | **0.0023** | -0.0320 | **14.00** | 437.00 | 0.0137 | **1.000** | Hou, Weng, 2010 |
| *Chrysolina aurichalcea* (Gebler in Mannerheim, 1825)* | Chrys | **0.0021** | -0.0145 | **6.88** | 474.84 | 0.0233 | **1.000** | Fujiyama, Harada, 1996 |
| *Chrysomela populi* (Linnaeus, 1758) | Chrys | **0.0032** | -0.0283 | **8.79** | 310.03 | 0.0446 | **0.999** | Gomi et al., 2005 |
| *Chrysomela vigintipunctata* (Scopoli, 1763) | Chrys | **0.0036** | -0.0320 | **8.91** | 278.85 | 0.0435 | **0.980** | Kutcherov, 2015 |
| *Chrysophtharta agricola* (Chapuis, 1877) | Chrys | **0.0028** | -0.0277 | **9.80** | 354.57 | 0.0335 | **1.000** | Nahrung et al., 2004 |
| *Entomoscelis americana* Brown, 1942* | Chrys | **0.0033** | -0.0310 | **9.32** | 300.75 | 0.0355 | **0.999** | Lamb, Gerber, 1985 |
| *Gastrolina depressa* Baly, 1859 | Chrys | **0.0048** | -0.0415 | **8.73** | 210.22 | 0.0556 | **1.000** | Kutcherov, 2016 |
| *Gastrophysa polygoni* (Linnaeus, 1758) | Chrys | **0.0032** | -0.0196 | **6.06** | 308.61 | 0.0452 | **0.999** | Hilterhaus, 1965 |
| *Gastrophysa viridula* (De Geer, 1775) | Chrys | **0.0036** | -0.0276 | **7.71** | 279.15 | 0.0475 | **0.998** | Kucherov, Kipyatkov, 2011 |
| *Leptinotarsa decemlineata* (Say, 1824) | Chrys | **0.0024** | -0.0241 | **10.04** | 416.77 | 0.0263 | **0.999** | Boukal et al., 2015 |
| *Microtheca ochroloma* Stål, 1860 | Chrys | **0.0031** | -0.0288 | **9.32** | 324.21 | 0.0329 | **1.000** | Manrique et al., 2012 |
| *Paropsis atomaria* Olivier, 1807 | Chrys | **0.0016** | -0.0134 | **8.30** | 619.19 | 0.0189 | **0.990** | Nahrung et al., 2008 |
| *Phaedon brassicae* Baly, 1874 | Chrys | **0.0030** | -0.0210 | **7.03** | 334.07 | 0.0388 | **0.995** | Wang et al., 2007 |
| *Phratora vulgatissima* (Linnaeus, 1758) | Chrys | **0.0018** | -0.0086 | **4.88** | 566.64 | 0.0234 | **0.997** | Pollard, 2014 |
| *Platyphora quadrisignata* (Germar, 1824)* | Chrys | **0.0024** | -0.0192 | **8.17** | 425.07 | 0.0278 | **1.000** | Schroder et al., 1994 |
| *Zygogramma bicolorata* Pallister, 1953 | Chrys | **0.0015** | -0.0124 | **8.22** | 661.78 | 0.0301 | **0.997** | Hasan, Shafiq Ansari, 2015 |
| *Crioceris asparagi* (Linnaeus, 1758) | Crioc | **0.0032** | -0.0345 | **10.81** | 313.47 | 0.0472 | **1.000** | Taylor, Harcourt, 1978 |
| *Crioceris quatuordecimpunctata* (Scopoli, 1763) | Crioc | **0.0037** | -0.0383 | **10.46** | 273.01 | 0.0496 | **0.996** | Chiba, Shinohe, 1975 |
| *Lema cyanella* Linnaeus, 1758 | Crioc | **0.0036** | -0.0436 | **12.10** | 277.70 | 0.0465 | **0.996** | Alec McClay, pers. comm. |
| *Lema diversa* Baly, 1873 | Crioc | **0.0033** | -0.0375 | **11.24** | 299.72 | 0.0509 | **0.996** | Cheng et al., 2001 |
| *Lilioceris faldermanni* (Guerin-Meneville, 1829) | Crioc | **0.0018** | -0.0097 | **5.39** | 558.36 | 0.0262 | **0.994** | Mojib et al., 2013 |
| *Oulema duftschmidi* (Redtenbacher, 1874) | Crioc | **0.0037** | -0.0522 | **14.29** | 273.68 | 0.0254 | **0.999** | Morlacchi et al., 2007 |
| *Oulema gallaeciana* (Heyden, 1870) | Crioc | **0.0032** | -0.0329 | **10.13** | 307.75 | 0.0321 | **0.998** | Ali et al., 1977 |
| *Oulema melanopus* (Linnaeus, 1758) | Crioc | **0.0024** | -0.0215 | **9.11** | 423.34 | 0.0257 | **1.000** | Ali et al., 1977 |
| *Oulema oryzae* (Kuwayama, 1931) | Crioc | **0.0032** | -0.0321 | **10.11** | 315.09 | 0.0314 | **0.997** | Syoji, 1972 |
| *Agasicles hygrophila* Selman et Vogt, 1971 | Galer | **0.0034** | -0.0442 | **12.98** | 293.77 | 0.0409 | **0.981** | Wu, 1997 |
| *Agelastica alni* (Linnaeus, 1758) | Galer | **0.0017** | -0.0154 | **8.83** | 571.97 | 0.0204 | **0.997** | Bukatina et al., unpublished |
| *Altica carduorum* (Guérin-Méneville, 1858) | Galer | **0.0025** | -0.0231 | **9.34** | 404.91 | 0.0239 | **0.999** | Wan et al., 1996 |
| *Altica litigata* Fall, 1910 | Galer | **0.0028** | -0.0245 | **8.92** | 363.54 | 0.0397 | **0.987** | Pettis, Braman, 2007 |
| *Argopistes coccinelliformis* Csiki, 1940 | Galer | **0.0023** | -0.0243 | **10.56** | 435.19 | 0.0274 | **0.996** | Tamura, Takeuchi, 1992 |
| *Cerotoma arcuata* (Olivier, 1791) | Galer | **0.0020** | -0.0213 | **10.45** | 490.22 | 0.0297 | **0.995** | Nava, Parra, 2003 |
| *Cerotoma ruficornis* (Olivier, 1791) | Galer | **0.0024** | -0.0328 | **13.90** | 423.57 | 0.0280 | **0.996** | Heyer et al., 1988 |
| *Cerotoma trifurcata* (Forster, 1771) | Galer | **0.0016** | -0.0148 | **9.33** | 631.55 | 0.0245 | **0.998** | McCreary, 2013 |
| *Diabrotica balteata* LeConte, 1865 | Galer | **0.0018** | -0.0178 | **9.64** | 541.37 | 0.0251 | **0.991** | Heyer, Cruz, 1983 |
| *Diabrotica barberi* R. Smith et Lawrence, 1967* | Galer | **0.0021** | -0.0219 | **10.33** | 470.98 | 0.0226 | **0.998** | Woodson, Jackson, 1988 |
| *Diabrotica speciosa* (Germar, 1824) | Galer | **0.0020** | -0.0216 | **10.65** | 493.26 | 0.0281 | **0.991** | Milanez, Parra, 2000 |
| *Diabrotica virgifera virgifera* LeConte, 1868* | Galer | **0.0027** | -0.0279 | **10.28** | 368.46 | 0.0291 | **0.986** | Jackson, Elliott, 1988 |
| *Diabrotica virgifera zeae* Krysan et Smith, 1980* | Galer | **0.0025** | -0.0283 | **11.32** | 400.62 | 0.0247 | **0.998** | Woodson, Chandler, 2000 |
| *Diorhabda elongata* Brullé, 1832 | Galer | **0.0026** | -0.0357 | **13.60** | 380.73 | 0.0300 | **0.998** | Herrera et al., 2005 |
| *Diorhabda tarsalis* Weise, 1889 | Galer | **0.0025** | -0.0268 | **10.87** | 406.30 | 0.0323 | **0.995** | Zhang et al., 2007 |
| *Galeruca sardoa* (Gené, 1839)* | Galer | **0.0022** | -0.0208 | **9.54** | 459.13 | 0.0257 | **0.997** | Uscidda, Crovetti, 1983 |
| *Galerucella birmanica* Jacoby, 1889 | Galer | **0.0031** | -0.0338 | **10.91** | 322.55 | 0.0437 | **1.000** | Chen, Chen, 2003 |
| *Galerucella calmariensis* (Linnaeus, 1767) | Galer | **0.0023** | -0.0218 | **9.32** | 428.31 | 0.0293 | **0.997** | McAvoy, Kok, 2004 |
| *Galerucella grisescens* (Joannis, 1865) | Galer | **0.0036** | -0.0425 | **11.76** | 276.38 | 0.0479 | **0.982** | Lin et al., 2002 |
| *Galerucella lineola* (Fabricius, 1781) | Galer | **0.0022** | -0.0154 | **7.04** | 456.77 | 0.0279 | **0.984** | Pollard, 2014 |
| *Galerucella nymphaeae* (Linnaeus, 1758) | Galer | **0.0031** | -0.0288 | **9.26** | 321.01 | 0.0326 | **0.999** | Tauber et al., 1996 |
| *Galerucella pusilla* (Duftschmid, 1825) | Galer | **0.0023** | -0.0210 | **9.07** | 431.99 | 0.0253 | **0.995** | McAvoy, Kok, 2004 |
| *Monolepta hieroglyphica* (Motchulsky, 1858)* | Galer | **0.0019** | -0.0215 | **11.10** | 517.28 | 0.0269 | **0.989** | Li et al., 2008 |
| *Ophraella communa* LeSage, 1986 | Galer | **0.0038** | -0.0474 | **12.48** | 263.23 | 0.0390 | **1.000** | Emura, 1999 |
| *Phyllotreta armoraciae* (Koch, 1803) | Galer | **0.0022** | -0.0238 | **10.63** | 446.99 | 0.0277 | **0.998** | Vig, 2003 |
| *Phyllotreta cruciferae* (Goeze, 1777) | Galer | **0.0024** | -0.0280 | **11.87** | 423.53 | 0.0310 | **0.996** | Kinoshita et al., 1979 |
| *Phyllotreta nemorum* (Linnaeus, 1758) | Galer | **0.0024** | -0.0264 | **11.03** | 417.99 | 0.0286 | **0.999** | Vig, 2003 |
| *Systena basalis* DuVal, 1856 | Galer | **0.0013** | -0.0154 | **12.14** | 787.48 | 0.0181 | **0.984** | Chiang Lok et al., 1987 |
| *Xanthogaleruca luteola* (Müller, 1766) | Galer | **0.0025** | -0.0288 | **11.68** | 405.53 | 0.0259 | **1.000** | King et al., 1985 |

**Total immature development, bad data**

| **Species** | **Sub- family** | **Regression coefficient** | **Intercept** | **Lower temperature threshold** | **Sum of degree-days** | **r^2^** | **Data source** |
| --- | --- | --- | --- | --- | --- | --- | --- |
| *Gratiana graminea* Klug, 1829 | Cass | **0.0032** | -0.0366 | **11.48** | 313.91 | **0.970** | Manrique et al., 2012 |
| *Plesispa reichei* (Chapuis, 1875) | Cass | **0.0013** | -0.0067 | **4.98** | 747.29 | **0.802** | Suwandharathne et al., 2011 |
| *Chrysolina aeruginosa* (Faldermann, 1835) | Chrys | **0.0010** | -0.0069 | **7.10** | 1026.51 | **0.967** | Wei et al., 2013 |
| *Chrysomela scripta* Fabricius, 1801 | Chrys | **0.0043** | -0.0453 | **10.54** | 232.59 | **0.962** | Burkot, Benjamin, 1979 |
| *Colaphellus bowringi* Baly, 1865 | Chrys | **0.0037** | -0.0346 | **9.40** | 271.21 | **0.955** | Xue et al., 2002 |
| *Gastrolina depressa* Baly, 1859 | Chrys | **0.0063** | -0.0767 | **12.21** | 159.31 | **0.974** | Meng et al., 2006 |
| *Gastrophysa atrocyanea* Motschulsky, 1860 | Chrys | **0.0036** | -0.0216 | **5.98** | 276.55 | **0.874** | Lee et al., 2002 |
| *Plagiodera versicolora* (Laicharting, 1781) | Chrys | **0.0039** | -0.0356 | **9.07** | 255.08 | **0.971** | Yang et al., 2006 |
| *Sclerophaedon orbicularis* (Suffrian, 1851) | Chrys | **0.0020** | -0.0079 | **3.97** | 500.17 | **0.953** | Fischer, 1985 |
| *Zygogramma bicolorata* Pallister, 1953 | Chrys | **0.0027** | -0.0314 | **11.49** | 366.34 | **0.972** | King, 2008 |
| *Zygogramma suturalis* (Fabricius, 1775) | Chrys | **0.0024** | -0.0280 | **11.45** | 408.32 | **0.975** | Igrc, 1988 |
| *Lema decempunctata* Gebler, 1830 | Crioc | **0.0023** | -0.0145 | **6.23** | 428.55 | **0.949** | Du et al., 2006 |
| *Lema scutellaris* (Kraatz, 1879) | Crioc | **0.0041** | -0.0544 | **13.25** | 243.38 | **0.959** | Zhang et al., 1995 |
| *Acalymma vittatum* (Fabricius, 1775) | Galer | **0.0020** | -0.0249 | **12.20** | 490.24 | **0.926** | Ellers-Kirk, Fleischer, 2006 |
| *Agasicles hygrophila* Selman et Vogt, 1971 | Galer | **0.0034** | -0.0421 | **12.32** | 292.39 | **0.947** | Stewart et al., 1999 |
| *Ophraella communa* LeSage, 1986 | Galer | **0.0030** | -0.0363 | **12.17** | 335.73 | **0.926** | Zhou et al., 2010 |
| *Pyrrhalta viburni* (Paykull, 1799)* | Galer | **0.0012** | -0.0092 | **7.56** | 819.61 | **0.926** | Weston, Diaz, 2005 |
