# Supplementary material for: Thermal reaction norms can surmount evolutionary constraints: comparative evidence across leaf beetle species
Source: Ecol Evol. 2016 Jun 12;6(14):4670–83. doi: 10.1002/ece3.2231 (PMC4979698; doi:10.1002/ece3.2231)
Supplement: Supplementary file 3 — Appendix S3 References for the dataset. [file ECE3-6-4670-s003.docx]

**Appendix 3
References for the dataset**

Ali, A.-W., Th. Wetzel, and W. Heyer. 1977. Ergebnisse von Untersuchungen über die Effektivtemperatursummen einzelner Entwicklungsstadien der Getreidehähnchen (*Lema* spp.). Arch. Phytopathol. Plant Prot. 13:425–433.

Aslan, I., R. Beenen, and H. Özbek. 2005. Biological aspects of *Galeruca circassica* Reitter, 1889 (Coleoptera: Chrysomelidae: Galerucinae) in relation to the weed *Cephalaria procera* Fish. and Lall. (Dipsacaceae) in Anatolia. Bonner zool. Beitr. 54:173–177.

Boukal, D. S., T. Ditrich, D. Kutcherov, P. Sroka, P. Dudová, and M. Papáček. 2015. Analyses of developmental rate isomorphy in ectotherms: introducing the Dirichlet regression. PLoS ONE e0129341.

Burkot, T. R., and D. M. Benjamin. 1979. The biology and ecology of the cottonwood leaf beetle, *Chrysomela scripta* (Coleoptera: Chrysomelidae), on tissue cultured hybrid Aigeiros (*Populus* × *euramericana*) subclones in Wisconsin. Can. Ent. 111:551–556.

Chen, S. K., and W. S. Chen. 2003. Preliminary studies on the ecology of *Galerucella nipponensis* (Laboissiere) (Coleoptera: Chrysomelidae). Res. Bull. Tainan Distr. Agric. Impr. St. 41:28–34.

Cheng, D.-W., X.-R. Zhang, J.-H. Liu, J.-L. Dong, and J.-R. Zhan. 2001. Effects of temperature of the development, food-take, reproduction of *Lema dilversa* Baly. Acta Ecol. Sinica 21:498–501.

Chevin, H. 1991. Contribution à la biologie des *Timarcha* (Col. Chrysomelidae). V. *Timarcha normanna* Reiche. L’Entomologiste 47:181–188.

Chevin, H. 1992. Contribution à la biologie des *Timarcha* (Col. Chrysomelidae). VI. *Timarcha maritima* Perris. L’Entomologiste 48:133–140.

Chiang Lok, M. L., W. Heyer, and B. Cruz. 1987. Influencia de la temperatura sobre el desarrollo de los estadios biológicos de *Systena basalis*. Ciencias Agric. 30:7–12.

Chiba, T., and S. Shinohe. 1975. Observations on the development of egg, larval and pupal stages of asparagus beetle, *Crioceris quatuordecimpunctata* Scopoli (Coleoptera, Chrysomlidae). J. North. Jap. Plant Protection Soc. 26:25–29.

Cristofaro, M., M. Yu. Dolgovskaya, A. S. Konstantinov, F. Lecce, S. Ya. Reznik, L. Smith, C. Tronci, and M. G. Volkovitsh. 2003. *Psylliodes chalcomerus* (Coleoptera: Chrysomelidae: Alticinae), a flea beetle candidate for biological control of yellow starthistle *Centaurea solstitialis*. Pages 75–80 *in:* J. M. Cullen, D. T. Briese, D. J. Kriticos, W. M. Lonsdale, L. Morin, and J. K. Scott (eds.) Proceedings of the XI International Symposium on Biological Control of Weeds. CSIRO Entomology, Canberra.

Deng, Y., S. Wu, and L. Li. 1999. Temperature effect on development and reproduction of Chinese cowpea weevil, *Callosobruchus chinensis* L. (Coleoptera: Bruchidae). Pages 125–127 *in:* Z. Jin, Q. Liang, Y. Liang, X. Tan, and L. Guan (eds.). Proceedings of the 7th International Working Conference on Stored-Product Protection. Vol. 1. Sichuan Publishing House of Science and Technology, Chengdu.

Diaz, R., W. A. Overholt, A. Samayoa, F. Sosa, D. Cordeau, and J. Medal. 2008. Temperature-dependent development, cold tolerance, and potential distribution of *Gratiana boliviana* (Coleoptera: Chrysomelidae), a biological control agent of tropical soda apple, *Solanum viarum* (Solanaceae). Biocontrol Sci. Tech. 18:193–207.

Doddala, P. R. C., S. A. Trewick, D. J. Rogers, and M. A. Minor. 2013. Predictive modelling of adult emergence in a polyphagous *Eucolaspis* (Chrysomelidae: Eumolpinae) leaf beetle. J. Econ. Entomol. 106:899–904.

Dominique, C. R., and W. N. Yule. 1983. Threshold temperature and thermal constant for egg development of northern corn rootworm, *Diabrotica longicornis* (Coleoptera: Chrysomelidae). Can. Ent. 115:1051–1052.

Dong, Y.-K., F.-S. Xue, and Z.-H. Li. 2007. Effect of temperature on development, survival and fecundity of *Colaphellus bowringi*. Chinese Bull. Entomol. 44: 524–526.

Du, Y.-N., Z.-S. Zhang, and R.-Q. Shen. 2006. Threshold temperature and effective accumulated temperature of *Lema decempunctata*. Chinese Bull. Entomol. 43:474–476.

El-Kifl, A. H., and M. M. Metwally. 1971. Biological and ecological studies on *Bruchidius incarnatus* Boh. [Coleoptera: Bruchidae]. Bull. Soc. ent. Egypte 55:141–162.

Ellers-Kirk, C., and S. J. Fleischer. 2006. Development and life table of *Acalymma vittatum* (Coleoptera: Chrysomelidae), a vector of *Erwinia tracheiphila* in cucurbits. Environ. Entomol. 35:875–880.

Emura, K. 1999. The ragweed beetle *Ophraella communa* LeSage (Coleoptera: Chrysomelidae) which injures harmful exotic plants. Plant Protection 53: 138–141.

Ferro, D. N., J. A. Logan, R. H. Voss, and J. S. Elkinton. 1985. Colorado potato beetle (Coleoptera, Chrysomelidae) temperature-dependent growth and feeding rates. Environ. Entomol. 14:343–348.

Fischer, R. 1985. Ökologische Untersuchungen an *Sclerophaedon orbicularis* (Suffr.) (Coleoptera: Chrysomelidae). Deutsche Entomol. Zeitschrift (neue Folge). 32:143–163.

Fornasari, L. 1995. Temperature effects on the embryonic development of *Aphthona abdominalis* (Coleoptera: Chrysomelidae), a natural enemy of *Euphorbia esula* (Euphorbiales: Euphorbiaceae). Environ. Entomol. 24:720–723.

Fujiyama, S., and K. Harada. 1996. Comparison of effects of temperature on the development of six geographic populations of *Chrysolina aurichalcea* (Mannerheim) (Coleoptera: Chrysomelidae). Jap. J. Appl. Entomol. Zool. 40:217-226.

Gandolfo, D., J. C. Medal, and J. P. Cuda. 2008. Effects of temperature on the development and survival of *Metriona elatior* (Coleoptera: Chrysomelidae) immatures. Florida Entomol. 91:491–493.

Giang, H. T. T., and S. Nakamura. 2009. The study on biological characteristics of *Brontispa longissima* (Gestro) (Coleoptera: Chrysomelidae). J. Sci. Dev. 7(Eng.Iss.2):159–164.

Golden, K. L. and L. J. Meinke. 1991. Immature development, fecundity, longevity, and egg diapause of *Diabrotica longicornis* (Coleoptera: Chrysomelidae). J. Kansas Ent. Soc. 64: 251–256.

Gomi, T., H. Hagihara, and T. Fukuda. 2005. Effect of temperature on development and reproduction in *Chrysomela populi* (Coleoptera: Chrysomelidae). Jap. J. Appl. Entomol. Zool. Chugoku Branch 47:1–6.

Gong, Z.-Q., J.-W. Wang et al. 2000. Studies on the development zero and effective accumulated temperature and the thermal constant of *Callosobruchus maculatus* Fabricius. Entomol. Knowl. 37:285–286.

Groden, E., and R. A. Casagrande. 1986. Population dynamics of the Colorado potato beetle, *Leptinotarsa decemlineata* (Coleoptera: Chrysomelidae), on *Solanum berthaultii*. J. Econ. Entomol. 79:91–97.

Guppy J. C., and D. G. Harcourt.1978. Effects of temperature on development of the immature stages of the cereal leaf beetle, *Oulema melanopus* (Coleoptera: Chrysomelidae). Can. Entomol. 110:257–263.

Hasan, F., and M. Shafiq Ansari. 2016. Temperature-dependent development and demography of *Zygogramma bicolorata* (Coleoptera: Chrysomelidae) on *Parthenium hysterophorus*. Ann. Appl. Biol. 168:81–92.

He, D., C. Tian, and H. Li. 1989. Constant temperature and fluctuating temperature effects on the development of *Diorhabda rybakowi* Weise. Acta Univ. Septentrionali Occident. Agric. 17:94–99.

Herrera, A. M., D. D. Dahlsten, N. Tomic-Carruthers, and R. I. Carruthers. 2005. Estimating temperature-dependent developmental rates of *Diorhabda elongata* (Coleoptera: Chrysomelidae), a biological control agent of saltcedar (*Tamarix* spp.). Environ. Entomol. 34:775–784.

Heyer, W., and B. Cruz. 1983. Influencia de la temperatura y la planta hospedera sobre el desarrollo de los estadios biológicos de *Diabrotica balteata* Lec. (Coleoptera: Chrysomelidae). Ciencias Agric. 17:31–40.

Heyer, W., M. L. Chiang Lok, and B. Cruz. 1988. Zum Einfluß der Temperatur und Wirtspflanze auf die Entwicklung von *Andrector ruficornis* (Oliv.) (Coleoptera: Chrysomelidae). Beitr. Ent. 38:183–188.

Hilbeck, A., and G. G. Kennedy. 1998. Effects of temperature on survival and preimaginal development rates of Colorado potato beetle on potato and horse-nettle: potential role in host range expansion. Entomol. Exp. Appl. 89:261–269.

Hilterhaus, V. 1965. Biologisch-ökologische Untersuchungen an Blattkäfern der Gattungen *Lema* und *Gastroidea* (Chrysomelidae, Col.). Z. Angew. Zool. 52:257–295.

Honěk A., V. Jarošik, and Z. Martinková. 2003. Effect of temperature on development and reproduction in *Gastrophysa viridula* (Coleoptera: Chrysomelidae). Eur. J. Entomol. 100:295–300.

Hou, Y., and Z. Weng. 2010. Temperature-dependent development and life table parameters of *Octodonta nipae* (Coleoptera: Chrysomelidae). Environ. Entomol. 39:1676–1684.

Howe, R. W., and J. E. Currie. 1964. Some laboratory observations on the rates of development, mortality and oviposition of several species of Bruchidae breeding in stored pulses. Bull. Entomol. Res. 55:437–477.

Hu, Z.-F., H.-C. Yu, W.-P. Sun, and F.-S. Xue. 2008. The developmental duration and biological characteristics of *Colaphellus bowringi* in Harbin. Chinese Bull. Entomol. 45: 909–912.

Igrc, J. 1989. Influence of temperature on development of *Zygogarmma suturalis* – an insect used to control Ambrosia artemisifolia. Proc. VII Int. Symp. Biol. Contr. Weeds. P. 613–621.

Ireson, J. E., D. A. Friend, R. J. Holloway, and S. C. Paterson. 1991. Biology of *Longitarsus flavicornis* (Stephens) (Coleoptera: Chrysomelidae) and its effectiveness in controlling ragwort (*Senecio jacobaea* L.) in Tasmania. J. Aust. ent. Soc. 30:129–141.

Jackson, J. J., and N. C. Elliott. 1988. Temperature-dependent development of immature stages of the western corn rootworm, *Diabrotica virgifera virgifera* (Coleoptera: Chrysomelidae). Environ. Entomol. 17:166–171.

Jacqmin, N., and G. Josens. 1977. Étude expérimentale de la crossaince et de la fécondité de *Gastrophysa viridula* DeGeer (Col., Chrys.) de la valée du Viroin (1). Annales Soc. R. Zool. Belg. 107:25–39.

King, H., 2008. Thermal physiology and predicted distribution of *Zygogramma bicolorata* (Chrysomelidae), a promising agent for the biological control of the invasive weed *Parthenium hysterophorus* in South Africa. MSc Thesis. University of KwaZulu-Natal.

King, J. E., R. G. Price, J. H. Young, L. J. Willson, and K. N. Pinkston. 1985. Influence of temperature on development and survival of the immature stages of the elm leaf beetle, *Pyrrhalta luteola* (Müller) (Coleoptera: Chrysomelidae). Environ. Entomol. 14:272–274.

Kinoshita, G. B., H. J. Svec, C. R. Harris, and F. L. McEwen. 1979. Biology of the crucifer flea beetle, *Phyllotreta cruciferae* (Coleoptera: Chrysomelidae) in Southwestern Ontario. Can. Ent. 111:1395–1407.

Kucherov, D. A., and V. E. Kipyatkov. 2011. Control of preimaginal development by photoperiod and temperature in the dock leaf beetle *Gastrophysa viridula* (De Geer) (Coleoptera, Chrysomelidae). Entomol. Rev. 91:692–708.

Kutcherov, D. A. 2015. Temperature-dependent development in *Chrysomela vigintipunctata* (Coleoptera: Chrysomelidae), a stenothermal early-season breeder. J. Therm. Biol. 53:9–14.

Kutcherov, D. A. 2016. Temperature effects on the development, body size, and sex ratio of the walnut leaf beetle *Gastrolina depressa* (Coleoptera: Chrysomelidae). J. Asia-Pacific Entomol. 19:153–158.

Kutcherov, D. A. E. B. Lopatina, and V. E. Kipyatkov. 2011. Photoperiod modifies thermal reaction norms for growth and development in the red poplar leaf beetle *Chrysomela populi* (Coleoptera: Chrysomelidae). J. Insect Physiol. 57:892–898.

Kutcherov, D. A., V. E. Kipyatkov, and E. B. Lopatina. 2014. Intrapopulational variation of thermal reaction norms for development in two species of leaf beetles (Coleoptera: Chrysomelidae). Vestnik SPbGU, Biology Series 4:10–21.

Lactin, D. J., and N. J. Holliday. 1992. Constant-temperature development rates of pre-imaginal Colorado potato beetles (*Leptinotarsa decemlineata* Say) (Coleoptera: Chrysomelidae) from Manitoba and British Columbia. Proc. Entomol. Soc. Manitoba 48:1–13.

Lamb, R. J., and G. H. Gerber. 1985. Effects of temperature on the development, growth, and survival of larvae and pupae of a north-temperate chrysomelid beetle. Oecologia 67:8–18.

Lee, S.-Y., Y.-H. Cho, and O.-S. Kwon. 2002. Effect of temperature on ecology of *Gastrophysa atrocyanea* (Col.: Chrysomelidae). Proceedings of the Joint Conference of Korean Society for Applied Entomology. P. 141.

Li, G.-W., J.-P. Zhang, J. Chen, and J. Liu. 2008. Threshold temperature and effective accumulated temperature of *Monolepta hieroglyphica*. Chinese Bull. Entomol. 45:621–624.

Lin, Z.-H., C.-C. Ji, Q.-J. Chen, Y.-Z. Zhang and J.-H. Chen. 2002. Studies of threshold temperature and effective accumulated temperature of *Galerucella grisescens*. Wuyi Sci. J. 18:80–83.

Logan, P. A., R. A. Casagrande, H. H. Faubert, and F. A. Drummond. 1985. Temperature-dependent development and feeding of immature Colorado potato beetles, *Leptinotarsa decemlineata* (Say) (Coleoptera: Chrysomelidae). Environ. Entomol. 14:275–283.

Loi, G., and A. Belcari. 1983. Influenza della temperatura sullo sviluppo degli stadi preimaginali del coleottero crisomelide *Chrysomela populi* L. Frustula Ent. N.S. 6:87–101.

Loi, G., and L. Fornasari. 1985. Influenza della temperatura sullo sviluppo dell'uovo di *Acanthoscelides obtectus* (Say) (Coleoptera: Bruchidae). Frustula Ent. N.S. 6:407–412.

Manrique, V., R. Diaz, C. Montemayor, D. Serrano, and R. D. Cave. 2012. Temperature-dependent development and cold tolerance of *Microtheca ochroloma* (Coleoptera: Chrysomelidae), a pest of cruciferous crops in the southeastern United States. Ann. Entomol. Soc. Am. 105:859–864.

Manrique, V., R. Diaz, and W. A. Overholt. 2012. Temperature-dependent development, diapause and cold tolerance of *Gratiana graminea*, a potential biological control agent of *Solanum viarum* in Florida, USA. BioControl 57:581–589.

Mathiasen, H., H. Sørensen, J. Bligaard, and P. Esbjerg. 2015. Effect of temperature on reproduction and embryonic development of the cabbage stem flea beetle, *Psylliodes chrysocephala* L., (Coleoptera: Chrysomelidae). J. Appl. Entomol. 139:600–608.

Maw, E. 1981. Biology of some *Aphthona* spp. (Col.: Chrysomelidae) feeding on *Euphorbia* spp. (Euphorbiaceae), with special reference to leafy spurge (*Euphorbia* sp. near *esula*). MSc. thesis. University of Alberta.

McAvoy, T. J., and L. T. Kok. 2004. Temperature dependent development and survival of two sympatric species, *Galerucella calmariensis* and *G. pusilla*, on purple loosestrife. BioControl 49:467–480.

McCreary, C. M., 2013. Development of a degree day model and economic thresholds for *Cerotoma trifurcata* (Coleoptera: Chrysomelidae) in Ontario. MSc. thesis. University of Guelph.

McGregor, P. G. 1989. Ecology of *Paropsis charybdis* Stål (Coleoptera: Chrysomelidae): a *Eucalyptus* defoliator in New Zealand. PhD thesis. Massey University.

Meng, L., J. Xu, and H.-B. Li. 2007. Dispersal and bionomics of the alien *Ophraella communa* in China mainland. Chinese J. Biol. Control 23:5–10.

Meng, Q.-Y., X.-H. Liu, G.-H. Yang, Y.-H. Pei, and X.-G. Sun. 2006. Developmental threshold temperature and effective accumulated temperature of *Gastrolina depressa*. Chinese Bull. Entomol. 43:848–850.

Milanez, J. M., and J. R. P. Parra. 2000. Biologia e exigências térmicas de *Diabrotica speciosa* (Germar) (Coleoptera:Chrysomelidae) em laboratório. An. Soc. Entomol. Brasil 29:23–29.

Mishra, P. R., S. K. Mukherjee, and D. Dash. 2012. Influence of environmental factors on the growth and development of groundnut bruchid *Caryedon serratus* (Olivier) in storage. Indian J. Entomol. 74:36–40.

Mojib, H. Z., M. Yousefpour, and M. N. Padasht. 2013. Biology of beetle *Lilioceris faldermanni* (Guerin) (Col.: Chrysomelidae), pest of Chel cheragh lily in different temperature conditions. J. Animal Research (Iranian J. Biol.) 26:344–354.

Morlacchi, P., L. Limonta, and J. Baumgártner. 2007. From a descriptive toward an explicative growth-based model on immature *Oulema duftschmidi* (Coleoptera: Chrysomelidae) development at different temperatures. Environ. Entomol. 36:245–255.

Nahrung, H. F., G. R. Allen, and V. S. Patel. 2004. Day-degree development and phenology modelling of the immature stages of *Chrysophtharta agricola* (Chapuis) (Coleoptera: Chrysomelidae), a pest of eucalypt plantations. Austr. J. Entomol. 43:177–183.

Nahrung, H. F., M. K. Schutze, A. R. Clarke, M. P. Duffy, E. A. Dunlop, and S. A. Lawson. 2008. Thermal requirements, field mortality and population phenology modelling of *Paropsis atomaria* Olivier, an emergent pest in subtropical hardwood plantations. Forest Ecol. Manag. 255:3515–3523.

Nava, D. E., and J. R. P. Parra. 2003. Biology of *Cerotoma arcuatus* (Coleoptera: Chrysomelidae) and field validation of a laboratory model for temperature requirements. J Econ. Entomol. 96:609–614.

Okamoto, C., K. Tsuda, D. Yamaguchi, S. Sato, R. W. Pemberton, and J. Yukawa. 2008. Life history and host specificity of the Japanese flea beetles *Trachyaphthona sordida* and *T. nigrita* (Coleoptera: Chrysomelidae), potential biological control agents against skunk vine, *Paederia foetida* (Rubiaceae), in the southeastern parts of the United States and Hawaii. Entomol. Sci. 11:143–152.

Omkar, S. Rastogi, and P. Pandey. 2008. Effect of temperature on development and immature survival of *Zygogramma bicolorata* (Coleoptera: Chrysomelidae) under laboratory conditions. Int. J. Trop. Insect Sci. 28:130–135.

Qin, Ch.-H., Ch. Chen, H. Wan, and J.-H. Li. 2009. Studies on biological characteristics of *Donacia provosti*. China Vegetables 24:57–61.

Pettis, G. V., and S. K. Braman. 2007. Effect of temperature and host plant on survival and development of *Altica litigata* Fall. J. Entomol. Sci. 42:66–73.

Pollard, C. 2014. A temperature-dependent development model for willow beetle species (Coleoptera: Chrysomelidae) in Ireland: simulation of phenology/voltinism in response to climate change. PhD Thesis. National University of Ireland, Maynooth.

Redžepagić, H., M. Maceljski, I. Balarin, and D. Čamprag. 1983. Biološka i ekološka istraživanja repine kaside (*Cassida nebulosa* L.) kao osnova za utvrđivanje suzbijanja. Zagreb. 166 p.

Schroder, R.F.W., B. Puttler, S.S. Izhevsky, and D. Gandolfo. Viviparity and larval development of *Platyphora quadrisignata* (Germar) (Coleoptera: Chrysomelidae) in Brazil. Coleopt. Bull. 48:237–243.

Simelane, D. O. 2007. Influence of temperature, photoperiod and humidity on oviposition and egg hatch of the root-feeding flea beetle *Longitarsus bethae* (Chrysomelidae: Alticinae), a natural enemy of the weed *Lantana camara* (Verbenaceae). Bull. Entomol. Res. 97:111–116.

Smith, A. M. 1992. Modeling the development and survival of eggs of pea weevil (Coleoptera: Bruchidae). Environ. Entomol. 21:314–321.

Smith, A. M., and S. A. Ward. 1995. Temperature effects on larval and pupal development, adult emergence, and survival of the pea weevil (Coleoptera: Chrysomelidae). Environ. Entomol. 24:623–634.

Stewart, C. A., R. B. Chapman, R. M. Emberson, P. Syrett, and C. M. A. Frampton. 1999. The effect of temperature on the development and survival of *Agasicles hygrophila* Selman & Vogt (Coleoptera: Chrysomelidae), a biological control agent for alligator weed (*Alternanthera philoxeroides*). New Zealand J. Zool. 26:11–20.

Stiefel, V., J. R. Nechols, and D. C. Margolies. 1997. Development and survival of *Anomoea flavokansiensis* (Coleoptera: Chrysomelidae) as affected by temperature. Environ. Entomol. 26:223–228.

Suwandharathne, N. I., L.C.P. Fernando, and J. P. Edirisinghe. 2011. Effect of temperature on the developmental stages of the two colour leaf beetle *Plesispa reichei*, a pest of coconut. Proceedings of the Peradeniya University Research Sessions, Sri Lanka 16:144.

Syoji, T. 1972. Effects of temperatures on the oviposition and the development of the rice leaf beetle, *Oulema oryzae* Kuwayama. J. North. Jap. Plant Protection Soc. 23:48–52.

Tamura, M., and M. Takeuchi. 1992. Threshold temperature and thermal constant for development of the fleabeetle, *Argopistes coccinelliformis* Csiki (Coleoptera: Chrysomelidae). Household Pests 14:82–87.

Tanaka, M., K. Yamawaki, and F. Nakasuji. 2002. Dynamic interaction between a leaf beetle, *Galerucella nipponensis* (Coleoptera: Chrysomelidae) and an aquatic plant, *Trapa japonica* (Trapaceae). I. Life history traits of *G. nipponensis*. Entomol. Sci. 5:187–192.

Tauber, C. A., M. J. Tauber, B. Gollands, R. J. Wright, and J. J. Obrycki. 1988. Preimaginal development and reproductive responses to temperature in two populations of the Colorado potato beetle (Coleoptera: Chrysomelidae). Ann. Entomol. Soc. Am. 81:755–763.

Tauber, M. J., C. A. Tauber, and J. R. Nechols. 1996. Life history of *Galerucella nymphaeae* and implications of reproductive diapause for rearing univoltine chrysomelids. Physiol. Entomol. 21:317–324.

Taylor, R. G., and D. G. Harcourt. 1978. Effect of temperature on developmental rate of the immature stages of *Crioceris asparagi*. Can. Ent. 110:57–62.

Uscidda, C., and A. Crovetti. 1983. Influenza della temperatura sullo sviluppo degli stadi preimaginali di *Galeruca sardoa* (Gené) (Coleoptera Chrysomelidae). Frustula Ent. N. S. 6:45–68.

Vig, K. 2003. Biology of *Phyllotreta* (Alticinae), with emphasis on Hungarian and middle European species. Pages 565–576 in: P. Jolivet, J. A. Santiago-Blay, and M. Schmitt (eds.). New Developments in the Biology of Chrysomelidae. SPB Acad. Publ., The Hague.

Walczak, F. 2005. Studies on leaf beetles (*Oulema* spp.) development for short-term forecasting – evaluation of effect of temperature and humidity on duration of egg incubation. J. Plant Protect. Res. 45:135–143.

Walgenbach, J. F., and J. A. Wyman. 1984. Colorado potato beetle (Coleoptera: Chrysomelidae) development in relation to temperature in Wisconsin. Ann. Entomol. Soc. Am. 77:604–609.

Wan, F.-H., P. Harris, L.-M. Cai, and M.-X. Zhang. 1996. Biology and ecology of *Altica carduorum* (Chrysomelidae: Coleoptera) from North-western China: a potential biocontrol agent for *Cirsium arvense* (Asteraceae) in Canada. Biocontrol Sci. Tech. 6:509–519.

Wang, X., X. Zhou, and C. Lei. 2007. Development, survival and reproduction of the Brassica leaf beetle, *Phaedon brassicae* Baly (Coleoptera: Chrysomelidae) under different thermal conditions. Pan-Pac. Entomol. 83:143–151.

Ward, R. H., and R. L. Pienkowski. 1978. Biology of *Cassida rubiginosa*, a thistle-feeding shield beetle. Ann. Ent. Soc. Am. 71:585–591.

Wei, S.-H., M.-M. Zhu, R. Zhang, W.-G. Huang, and Z. Yu. 2013. Effects of temperature on the development and reproduction of *Chrysolina aeruginosa*. Acta Entomol. Sinica 56:1004–1009.

Woodson, W. D., and L. D. Chandler. 2000. Effects on development of immature Mexican corn rootworm (Coleoptera: Chrysomelidae). Ann. Ent. Soc. Am. 93:55–58.

Woodson, W. D., and J. J. Jackson. 1996. Developmental rate as a function of temperature in northern corn rootworm (Coleoptera: Chrysomelidae). Ann. Ent. Soc. Am. 89:226–230.

Wu, Z. 1997. Effect of temperature on the growth and development of *Agasicles hgrophila*. Chinese J. Appl. Ecol. 8:181–184.

Xi, J.-H., H.-Y. Pan, and Y.-J. Chen. 2000. Developmental threshold temperature and the effective accumulated temperature of *Gastrophysa polygoni* (Linnaeus). Entomol. Knowl. 37:283–284.

Xue, F.-S., A.-Q. Li, X.-F. Zhu, A.-L. Gui, P.-L. Jiang, and X.-F. Liu. 2002. Diversity in life history of the leaf beetle, *Colaphellus bowringi* Baly. Acta Entomol. Sinica 45:494–498.

Yang, Z.-D., X.-Q. Tian, and B.-G. Zhao. 2006. Threshold and effective accumulative temperature for the development of *Plagiodera versicolora*. J. Beijing Forestry Univ. 28:139–141.

Yoshizaki, M., and A. Ozawa. 2009. Reproductive traits of tea leaf beetle, *Demotina fasciculata* Baly (Coleoptera, Chrysomelidae). I. Sex ratio in tea fields, oviposition sites in a cage and effective accumulated temperature of egg development. Annual Report of the Kanto-Tosan Plant Protection Society 56:111–113.

Zeiss, M. R., K. J. Koehler, and L. P. Pedigo. 1996. Degree-day requirements for development of the bean leaf beetle (Coleoptera: Chrysomelidae) under two rearing regimes. J. Econ. Entomol. 89:111–118.

Zhang, X.-R., P. Shen, and R.-L.Wang. 1995. A study on the developmental threshold temperature and thermal constant of *Lema scutellaris* Kraatz. Entomol. Knowl. 32:359–360.

Zhang, Z., C. Yang, L.-I. Gao, and Y. Li. 2007. Life table of the laboratory population of *Diorhbda tarsalis* Weise at different temperatures. Acta Phytoph. Sin. 34:5–9.

Zheng, F.-S., Y.-Z. Du, Z.-J. Wang, and J.-J. Xu. 2008. Effect of temperature on the demography of *Galerucella birmanica* (Coleoptera: Chrysomelidae). Insect Science 15:375–380.

Zhong, Y., H. Li, K. Liu, H. Wen, Q. Jin, and Z. Peng. 2005. Effects of temperature on *Brontispa longissima* population growth. Chinese J. Appl. Ecol. 16:2369–2372.

Zhou, Z.-S., J.-Y. Guo, H.-S. Chen, and F.-H. Wan. 2010. Effects of temperature on survival, development, longevity, and fecundity of *Ophraella communa* (Coleoptera: Chrysomelidae), a potential biological control agent against *Ambrosia artemisiifolia* (Asterales: Asteraceae). Environ. Entomol. 39:1021–1027.

Zhou, Z.-X., J.-C. Luo, H.-P. Lü, and W.-C. Guo. 2010. Influence of temperature on development and reproduction of experimental populations of the Colorado potato beetle, *Leptinotarsa decemlineata* (Say) (Coleoptera: Chrysomelidae). Acta Entomol. Sinica 53:926–931.
